# Peroxisomal Degradation Correlates with the Progression of Kidney Injury in a UUO Mouse Model

**DOI:** 10.3390/biology15130996

**Published:** 2026-06-25

**Authors:** Jinhwi Kim, Hyunsoo Kim, Arun Chhetri, Laxman Manandhar, Gyuho Jang, Channy Park, Raekil Park

**Affiliations:** 1 Department of Biomedical Science and Engineering, Gwangju Institute of Science and Technology, Gwangju 61005, Republic of Korea; nukedata@gist.ac.kr (J.K.); ruyhyunsookim@gm.gist.ac.kr (H.K.); chhetri5@gm.gist.ac.kr (A.C.); laxmanandhar@gm.gist.ac.kr (L.M.); janggh-9008@naver.com (G.J.); channypark@gist.ac.kr (C.P.); 2 Department of Colorectal Surgery, Wonkwang University Hospital, Iksan 54538, Republic of Korea

**Keywords:** peroxisome, autophagy, UUO, renal damage, ROS

## Abstract

Background: The kidney is an organ rich in peroxisomes, which play a pivotal role in fatty acid oxidation and ROS decomposition. Importantly, peroxisomal dysfunction contributes to the development and progression of various renal diseases. Therefore, we aimed to elucidate whether peroxisomes affect renal damage and fibrosis over time using a unilateral ureteral obstruction (UUO) mouse model. Methods: Expression levels of peroxisome-related factors and ROS- and hypoxia-related genes in UUO mice were measured in a time-dependent manner. Results: UUO led to renal damage and fibrosis progression over time; it significantly increased the protein expression levels of ATG5 and ATG7, while it decreased PMP70 and PEX14 protein expression. In particular, UUO increased the protein expression level of pexophagy receptor NBR1. Although the number of peroxisomes decreased, the protein expression levels of peroxisomal biogenesis-related proteins such as PEX11b, PEX16, and PEX19 remained constant. Decreased lipid metabolism due to reductions in ACOX1, DBP, and catalase caused by UUO and increased ROS production through peroxisomal degradation and mitochondrial antioxidant enzyme dysfunction were observed. Additionally, HIF-1α protein levels gradually increased in the UUO mice, whereas those of HIF-2α initially increased and then decreased. Conclusions: UUO is characterized by a progressive, chronological reduction in peroxisomal markers. Our findings indicate that peroxisomal degradation and associated metabolic dysfunction are tightly correlated with the progression of kidney injury and fibrosis, suggesting a potential involvement of compromised peroxisomal homeostasis in renal pathogenesis rather than proving a direct causal mechanism. Maintaining peroxisomal quality control may nevertheless represent a potential therapeutic avenue for chronic kidney disease.

## Simple Summary

Renal fibrosis is a common consequence of various chronic kidney diseases and ultimately leads to kidney failure. Because the pathogenesis of renal fibrosis is complex, finding effective treatments remains a major challenge. The kidney is an organ rich in peroxisomes, which play a pivotal role in fatty acid oxidation and reactive oxygen species decomposition. Peroxisomal dysfunction may contribute to the development and progression of various renal diseases, including acute kidney injury and chronic kidney diseases. However, research on the mechanisms of kidney damage has primarily focused on mitochondria. In this study, we demonstrate that autophagic degradation of peroxisomes induced by a unilateral ureteral obstruction (UUO) is a key process in mouse kidney injury. Therefore, our findings provide valuable insights into the underlying mechanisms of renal fibrosis and can contribute to the development of novel treatments for renal diseases.

## 1. Introduction

The kidney is an organ rich in peroxisomes, which play a pivotal role in fatty acid oxidation (FAO) and decomposition of reactive oxygen species (ROS) [1]. Notably, FAO is the major energy source for renal proximal tubular epithelial cells [2], and impaired FAO and ROS scavenging induce lipid accumulation and subsequent cellular oxidative stress, which is further exacerbated by peroxisomal dysfunction [3]. Peroxisomes are also involved in cell death, innate immunity, and inflammatory responses [4]. Thus, acute kidney injury (AKI) and chronic kidney disease (CKD) can be caused and progressed by peroxisomal dysfunction [4,5].

One of the repercussions of chronic kidney disease (CKD) is renal fibrosis, which can lead to kidney failure. Due to the complex pathophysiology of renal fibrosis, finding effective treatments remains a significant challenge. CKD represents a critical public health problem, and is associated with high medical costs and a poor prognosis [4]. It is associated with a multifaceted disease process that leads to irreversible and structural changes in the kidney over time [4]. Several studies have shown that renal tissue hypoxia is closely linked to the progression of CKD, and is considered a common pathway that can initiate this process [6,7,8,9]. Furthermore, peroxisomal dysfunction links renal hypoxia and fibrosis through causing metabolic stress and ROS buildup, leading to lipid accumulation, and severe oxidative damage [1]. In addition, renal fibrosis is chronic, and serves as the common final pathway in nearly all chronic renal failure states, regardless of etiology [4]. Various animal models have been developed to elucidate the underlying mechanisms of renal fibrosis and evaluate the effectiveness of therapeutic interventions. In these models, surgical procedures such as unilateral ureteral obstruction (UUO) are widely used to induce renal fibrosis [10]. However, effective treatments to slow the progression of renal fibrosis and prevent complications associated with CKD are very limited [4]. Therefore, further research into the molecular mechanisms underlying the progression of renal fibrosis is urgently needed.

Homeostasis of peroxisome biogenesis and degradation is maintained through peroxisomal proteins [11]. The optimal functioning and quantity of peroxisomes are regulated in response to environmental changes, with both peroxisomal biosynthesis and pexophagy regulating the number of peroxisomes [12]. Pexophagy is a selective autophagy pathway through which most peroxisomes are removed. NBR1 and p62 (SQSTM1) are key autophagy receptors/adapters that link ubiquitinated proteins of the peroxisomal membrane with autophagosomes [13]. It is tightly regulated by various signaling pathways and can be activated above basal levels by several factors, including cellular stress, changes in nutrient levels, and environmental stimuli. In particular, the accumulation of dysfunctional peroxisomes leads to oxidative stress and metabolic disorders [14]. Moreover, peroxisomes are linked to mitochondria in FAO and ROS removal [1,3]. Most recent studies on the molecular mechanisms of kidney injury have focused on mitochondria; however, the role of peroxisomes has often been overlooked [3,15]. In this study, we aimed to investigate the mechanism by which peroxisomes influence renal damage and fibrosis induced by UUO. Our results demonstrated that peroxisomal degradation via hypoxia and ROS is a critical process in renal injury and fibrosis in the UUO mouse model.

## 2. Materials and Methods

### 2.1. Animals

Eight-week-old male C57BL/6J mice were purchased from Damul Science (Daejeon, Republic of Korea) and housed in a temperature-controlled facility (22 ± 1 °C) with a 12 h light/dark cycle, with food and water available ad libitum. Mice were randomly assigned to the experimental groups. To ensure biological reproducibility, the animal experiments were conducted across multiple independent chronological rounds (an initial block of 5 mice per group, followed by subsequent replicate cohorts). After accounting for minimal surgical mortality, a cumulative sample size of *n* = 9 individual mice per group (independent biological replicates) was successfully achieved and utilized for downstream Western blot and histological analyses. For specific qPCR transcripts where tissue availability was constrained, a randomly selected subset of *n* = 5 independent biological samples per group was utilized. All samples were processed and analyzed individually without physical pooling. All protocols and procedures for mice were approved by the Institutional Animal Care and Use Committee of the Gwangju Institute of Science and Technology (GIST-2018-054, Gwangju, Republic of Korea). Mice were sacrificed at designated time points to monitor the chronological progression of the disease model. For the baseline histological, phenotypic, and fibrotic analyses, kidney tissues were harvested at days 1, 3, 7, 10, and 14 post-surgeries. To capture the early-to-intermediate kinetic transitions of autophagy-mediated degradation pathways, additional early-phase cohorts were monitored at days 2 and 5. Tissues collected at these precise kinetic intervals (days 2, 5, 7, and 10) were selectively allocated for NBR1-related autophagic experiments. Animals were administered preoperative analgesics (buprenorphine 0.05 mg/kg and carprofen 5 mg/kg) via subcutaneous injection and anesthetized using 5% isoflurane (Hana Pharma Co., Ltd., Seoul, Republic of Korea) at a flow rate of 2 L/min. After anesthesia induction, 2–3% isoflurane was maintained at a flow rate of 2 L/min [16]. Depth of anesthesia was monitored via heart rate, respiratory rate, and limb reflexes induced by periodically pinching the paws and tail. Rapid recovery occurred within 2–5 min after the anesthetic was switched off following surgery. For the UUO model, a mid-abdominal incision was made followed by isolation of the left ureter. Then, the ureter was obstructed by a 4-0 silk suture by two-point ligation in the mid-ureter. After surgery, the incision site was sutured and the mice were raised for each indicated period. Afterward, mice were anesthetized with ketamine/xylazine and sacrificed to collect tissue and blood samples, and the occurrence of hydronephrosis was visually examined to confirm whether the ureteral obstruction was successful [17]. In the following biochemical experiment, an obstructed kidney on day 0, baseline, was used as a control for comparison. Serum was extracted by centrifuging blood (1000× *g*, 10 min). The concentrations of BUN, creatinine, and NGAL were measured at the Laboratory Animal Resource Center (GIST, Gwangju, Republic of Korea). After cutting the kidney tissue in the coronal plane, the pieces were immediately frozen in liquid nitrogen and stored at −80 °C.

### 2.2. Histology

Cryosections were prepared as described previously [18]. Kidney sections were stained with hematoxylin and eosin (H&E) and Sirius Red. For H&E staining, the tissue was first rinsed with physiological saline and then fixed in a 4% paraformaldehyde solution for 30–50 min. After fixation, the tissue underwent washing, dehydration, clearing, wax infiltration, embedding, and section preparation. Tissue sections were placed on glass slides and dried in a 45 °C incubator. After deparaffinization with xylene, the sections were rehydrated stepwise using alcohol solutions in order from high to low concentration, followed by washing with distilled water for 2 min. The sections were stained with hematoxylin for 5 min and washed with running water to remove excess stain; subsequently, they were differentiated in a 1% ethanol hydrochloride solution for 3 s and stained with eosin for approximately 2 min. Finally, the sections were dehydrated, cleared, and mounted. Sirius Red staining was performed by incubating slides in 0.1% Sirius Red F3B (Sigma-Aldrich, Seoul, Republic of Korea) for 1 h, washing twice in acidified water, dehydrating thrice in 100% ethanol, and then clearing in xylene. Images were captured with an OLYMPUS BX-51 microscope (cellSense Standard ver.1.18, Olympus, Tokyo, Japan) and analyzed with Image J software (ImageJ 1.54p).

### 2.3. Protein Preparation and Western Blotting

Western blotting was performed as described previously [19]. After washing the tissues with PBS, they were lysed in RIPA buffer using prefilled tube kits/Triple-Pure High Impact Zirconium Beads 3.0 mm (Benchmark Scientific, Sayreville, NJ, USA). Tissues were then homogenized using a BeadBug6 bead homogenizer (Benchmark Scientific) and their protein content was measured using the Bio-Rad Protein Quantitation Kit (Bio-Rad Korea, Seoul, Republic of Korea). The PageRuler™ Pre-Stained Protein Ladder (Thermo Scientific, Seoul, Republic of Korea) was used as a protein size marker. Equal amounts (20 μg) of each protein extract were loaded, separated by SDS-PAGE, transferred to nitrocellulose membranes (0.45 µm pore size), washed with 1× TBS-T, and blocked with a solution of 5% skim milk in 1× TBS-T for 1 h at room temperature. The membranes were incubated overnight at 4 °C with primary antibodies in 3% BSA solution. Then, they were washed three times for 5 min each with 1× TBS-T solution, and then incubated with secondary antibodies in 3% skim milk solution for 1 h at room temperature. The membranes were then washed three times for 5 min each with 1× TBS-T solution, placed in enhanced chemiluminescence solution (Thermo Scientific), and visualized using a ChemiDoc™ Touch Imaging System (Bio-Rad). GAPDH was selected and utilized as the internal loading control, as its expression stability across all experimental groups (control and UUO-treated time points) was experimentally confirmed prior to relative normalization, displaying higher consistency under fibrotic stress than structural markers [20]. Protein expression levels were quantified by measuring the intensity of protein bands in Western blots using ImageJ, and the measurement process was conducted in a non-blind manner. A list of antibodies is presented in Supplementary Table S1.

### 2.4. Immunofluorescence

Immunofluorescence (IF) was performed as described previously [19]. The tissue on the slide was permeabilized with 0.5% Triton X-100 for 5 min at room temperature, washed three times with 1× PBS, and blocked with 3% BSA solution diluted in PBS for 1 h. Primary antibodies diluted in 3% BSA were reacted overnight at 4 °C, and the slides were washed with 1× PBS and incubated with secondary antibodies for 1 h at room temperature. After washing with 1× PBS, the slides were observed under a confocal microscope (IX73, Olympus, Tokyo, Japan), with the results analyzed using Olympus cellSens Standard software (ver. 4.2). For quantification of IF staining, 5–10 non-overlapping fields were randomly selected from each section. Image analysis was performed using ImageJ software by setting consistent brightness and contrast thresholds.

### 2.5. Measurement of Co-Localization of LAMP1 and PMP70

To quantify the signals in renal tubules, high-magnification (1000×) IF images were randomly captured from the stained kidney sections. For analysis using ImageJ software, a minimum of six random, non-overlapping high-power fields (HPFs) were evaluated per individual animal sample (*n* = 5 mice per experimental group) to ensure statistical representation. To adhere to strict guidelines for biological experimental units, the values obtained from the multiple HPFs of a single mouse were pooled and averaged to generate a single representative data point per animal, thereby avoiding artificial inflation of the sample size. The degree of spatial overlap between lysosomes and peroxisomes was quantified using the Just Another Colocalization Plugin (JACoP v2.0) within the ImageJ ecosystem. Co-localization efficiency was determined by calculating Manders’ overlap coefficient (OC), which ranges continuously from 0 (indicating completely non-overlapping spatial distributions) to 1 (representing absolute 100% co-localization between the two fluorophores) [21]. Crucially, the OC was mathematically assessed to specifically measure the fraction of the peroxisomal marker PMP70 fluorescent signal that directly overlapped with the lysosomal marker LAMP1 signal, thereby providing a quantitative index of pexophagy induction.

### 2.6. Quantitative Real-Time PCR (qPCR)

For qPCR assays, a representative subset of *n* = 5 individual biological replicates (randomly selected from the final cohort of 9) was utilized for statistical analysis. Total RNA from tissue was prepared using TRIzol reagent (Invitrogen, Carlsbad, CA, USA), according to the manufacturer’s instructions. RNA purity was evaluated by analyzing absorbance ratios using a NanoDrop One (Thermo Scientific), with a focus on the A260/A280 ratio (range 1.8–2.1) for detecting protein contamination and the A260/A230 ratio (range 1.8–2.2) for detecting organic compound contamination. A reverse transcription kit (HK Genomics, Daejeon, Republic of Korea) was used to transcribe cDNA. Real-time qPCR was performed with a cDNA template using a light cycler system with SYBR green PCR master mix (Applied Biosystems, Austin, TX, USA). Relative expression analysis with the housekeeping gene 36B4 as an internal control confirmed stable Ct values across experimental groups. Primer sequences are presented in Supplementary Table S2.

### 2.7. Lipid Extraction and Gas Chromatography–Mass Spectrometry (GC-MS) Analysis

Lipids were extracted using the Bligh and Dyer method, and the extracted lipids were diluted in 200 μL of hexane prior to GC-MS analysis [22]. Briefly, tissues were homogenized in PBS, and the protein concentration was measured using the Bradford assay. Homogenates were added to 1.9 mL extraction solution (a chloroform: methanol volumetric ratio of 1:2) and 40 ng C23:0 as an internal standard, and then vortexed for 2 min. Samples were then added to chloroform (625 μL) and vortexed for 30 s, followed by rinsing with deionized water (625 μL) for 30 s. Then, samples were centrifuged at 3260 rpm for 10 min, and the lower phase (organic) layers were collected and transferred to glass tubes. The extracts were dried under a stream of nitrogen at 40 °C. After cooling, the samples were subsequently dissolved in toluene (200 μL) and mixed with methanol (1.5 mL) and acid chloride (300 μL; 8%) for the conversion of lipid to fatty acid methyl esters (FAMEs). Samples were then cooled and mixed with hexane (1 mL) and deionized water (1 mL). The hexane layers were collected and transferred to new tubes, and extraction with hexane was repeated two more times to optimize the collection of FAMEs. Finally, the hexane layer-pooled FAMEs were dried under nitrogen, dissolved in 100% hexane (100 μL), and transferred to a GC vial for analysis with GCMS-QP2020 (Shimadzu, Kyoto, Japan). GC parameters were as follows: injection temperature 230 °C, pressure 49.7 kPa, total flow rate 24.0 mL/min, column flow rate 1.00 mL/min, linear velocity 36.1 cm/sec, and purge flow rate 3.0 mL/min. MS was performed at an ion source temperature of 200 °C and an interface temperature of 300 °C. The peak areas of target lipid species, including plasmalogens, were normalized to the peak area of the C23:0 internal standard to correct for extraction efficiency, and final quantitative values were strictly normalized against the total protein content of each sample (expressed as concentration per milligram of protein) and the ratio to the control was calculated.

### 2.8. Statistical Analysis

Statistical analysis was performed using GraphPad Prism version 9.0 (GraphPad Software, San Diego, CA, USA). Data are presented as mean ± standard deviation (SD). For comparisons among multiple experimental groups, a standard one-way analysis of variance (ANOVA) was performed, followed by Tukey’s honest significant difference post hoc test for multiple pairwise comparisons. To ensure statistical transparency and rigor, exact *p*-values are reported within the figures and figure legends where applicable, rather than relying solely on asterisks. A *p*-value of less than 0.05 was considered statistically significant (* *p* < 0.05, ** *p* < 0.01, *** *p* < 0.001).

## 3. Results

### 3.1. UUO Induces Kidney Damage and Fibrosis

**Figure 1:**
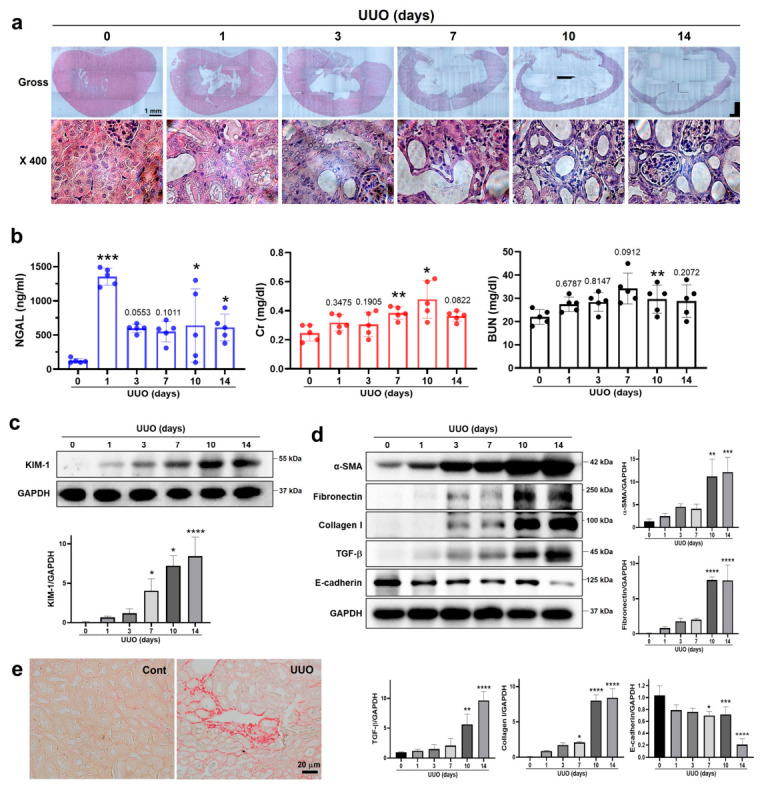
Kidney damage and fibrosis markers in the UUO models. UUO was performed in mice for indicated periods. Blood and kidney tissue were harvested in a different time period. (**a**) H&E staining. Scale bar = 1 mm. (**b**) Serum levels of NGAL, creatinine (Cr), and BUN. For the biochemical analysis of serum markers, a total of five independent biological samples per group were utilized for statistical analysis. Data are expressed as mean ± SD (*n* = 5 individual samples per group). Statistical analysis was performed via one-way ANOVA followed by Tukey’s post hoc test (NGAL, *p* = 0.0553 for control vs. 3 d and *p* = 0.1011 for control vs. 7 d; Cr, *p* = 0.3475 for control vs. 1 d, *p* = 0.1905 for control vs. 3 d, and *p* = 0.0822 for control vs. 14 d; BUN, *p* = 0.6787 for control vs. 1 d, *p* = 0.8147 for control vs. 3 d, *p* = 0.0912 for control vs. 7 d, and *p* = 0.2072 for control vs. 14 d; * *p* < 0.05, ** *p* < 0.01, *** *p* <0.001, compared with the control group). (**c**,**d**) Western blot and quantification of KIM-1, α-SMA, fibronectin, collagen I, TGF-β, and E-cadherin antibodies. GAPDH was used as a loading control. * *p* < 0.05, ** *p* < 0.01, *** *p* <0.001, **** *p* < 0.0001, compared with the control group. (**e**) Sirius Red staining, scale bar = 20 mm. Data are expressed as mean ± SD (*n* = 9 individual mice per group). Statistical analysis was performed via one-way ANOVA followed by Tukey’s post hoc test (* *p* < 0.05, ** *p* < 0.01, *** *p* <0.001, **** *p* < 0.0001, compared with the control group).

The success of ureteral obstruction by UUO was confirmed by direct visual inspection of the kidney at the time of sacrifice, and severe hydronephrosis was observed over time [17]. To evaluate the longitudinal progression of renal injury in the UUO model, biochemical analyses were performed against the day 0 obstructed kidney (baseline) as the primary control group. Although temporally matched, sham-operated controls were harvested at each experimental time point. They were excluded from the final biochemical timeline; the existing literature demonstrates that unilateral obstruction exerts negligible systemic or pathological effects on baseline renal parameters over a 14-day period. First, UUO progression in the mouse model, H&E staining was performed at 0, 1, 3, 7, 10, and 14 days after surgery. As shown in Figure 1a, the tubules were observed to be dilated at surgery after 3 days. At 7 days, the glomerulus was found to be distorted, and the epithelium had become thin compared to that in the control. In addition, infiltration of connective tissue was observed after 10 days. All of these findings are typical of the conventional phenotype of UUO progression [23]. Next, kidney damage markers, including neutrophil gelatinase-associated lipocalin (NGAL), creatinine (Cr), blood urea nitrogen (BUN), and kidney injury molecule 1 (KIM-1) were investigated at 0, 1, 3, 7, 10, and 14 days post-surgery [24]. Serum NGAL level peaked 1 day after UUO and exhibited a sustained increase compared to the control. The BUN and Cr serum levels, as well as the protein expression of KIM-1, increased in a time-dependent manner after UUO (Figure 1b,c). After UUO, fibrosis-promoting markers including α-SMA, fibronectin, collagen I, and TGF-β were significantly increased in the kidney tissue, while the epithelial marker E-cadherin decreased (Figure 1d). Furthermore, Sirius Red staining showed that collagen had accumulated in the renal tissue after UUO, which represented fibrosis (Figure 1e). These data demonstrated that UUO increased renal damage and fibrosis in a time-dependent manner in the mice.

### 3.2. UUO Decreases Kidney Peroxisome Number and Function

**Figure 2:**
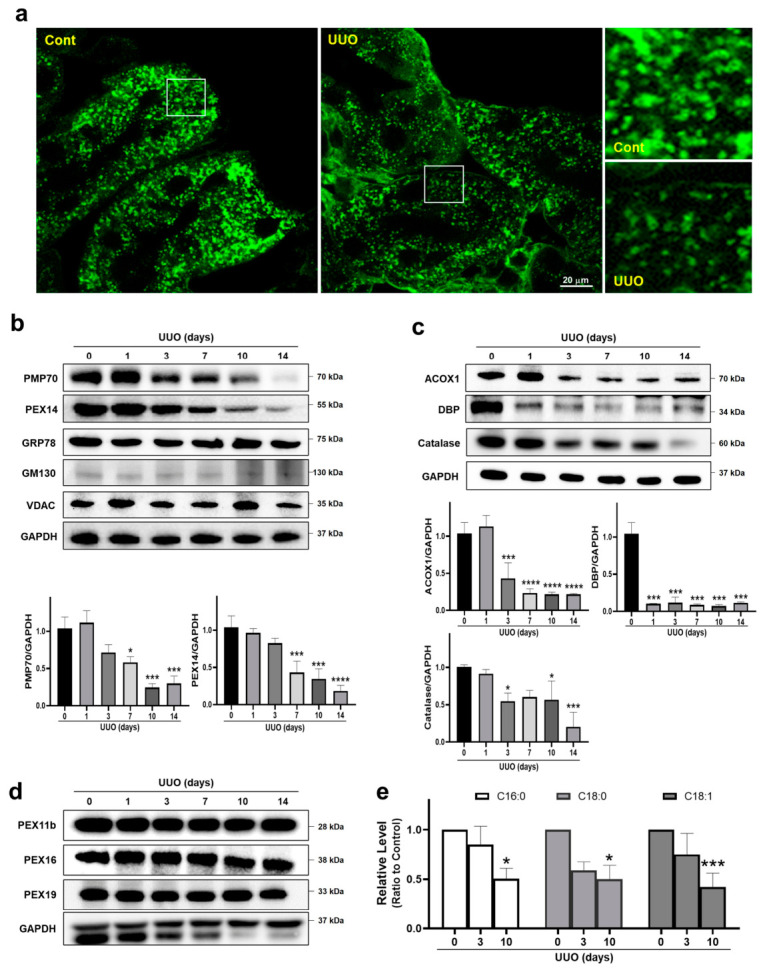
Markers of peroxisome number and function in the UUO models. UUO was performed in mice for indicated periods and kidney tissue was harvested. (**a**) Immunofluorescence staining for PMP70, scale bar = 20 mm. (**b**–**d**) Western blot and quantification of PMP70, PEX14, GRP78, GM130, VDAC, ACOX1, DBP, catalase, PEX11b, PEX16, and PEX19 antibodies. The complete images of the Western blots are provided in the Supplementary Materials. GAPDH was used as a loading control. (**e**) Plasmalogen levels. Data are expressed as mean ± SD (*n* = 9 individual mice per group). Statistical analysis was performed via one-way ANOVA followed by Tukey’s post hoc test (* *p* < 0.05, *** *p* <0.001, **** *p* < 0.0001, compared with the control group).

Maintaining optimal peroxisome number and function is important for homeostasis in response to environmental changes [25]. We found a decrease in peroxisome number in the renal tissue 7 days after UUO compared with the control group, as indicated by the quantification of PMP70 puncta per high-power field (Figure 2a). In addition, the expression of peroxisomal membrane markers, such as PMP70 and PEX14, was also decreased in a time-dependent manner, whereas endoplasmic reticulum (ER), Golgi apparatus, and mitochondrial proteins—such as GRP78, GM130, and VDAC—were not changed (Figure 2b). Furthermore, peroxisome-specific enzymes that indirectly indicated peroxisome function, such as catalase, acyl-CoA oxidase 1 (ACOX1), and peroxisomal D-bifunctional protein (DBP), also declined with UUO progression (Figure 2c). However, the expression levels of the selected peroxisomal biogenesis-related proteins evaluated in this study, including PEX11, PEX16, and PEX19, did not show marked alterations following UUO (Figure 2d).

We also detected the levels of plasmalogens, including C16:0, C18:0, and C18:1, in the renal tissue of the UUO mice. Renal tissue analysis of mice with UUO revealed significant changes in plasmalogen levels (particularly those of C16:0, C18:0, and C18:1), which, importantly, can indicate changes in lipid metabolism during renal fibrosis and oxidative stress [26]. Plasmalogen biosynthesis begins in peroxisomes and is completed in the ER [25]; deficiencies in this process are often due to peroxisome dysfunction [27]. The plasmalogen levels were significantly reduced 10 days after UUO (Figure 2e). These results cautiously indicate that peroxisomal deterioration in UUO may be associated with the degradation of existing peroxisomes, rather than a primary impairment of these specific biogenesis pathways.

### 3.3. UUO Induces Autophagic Process

**Figure 3:**
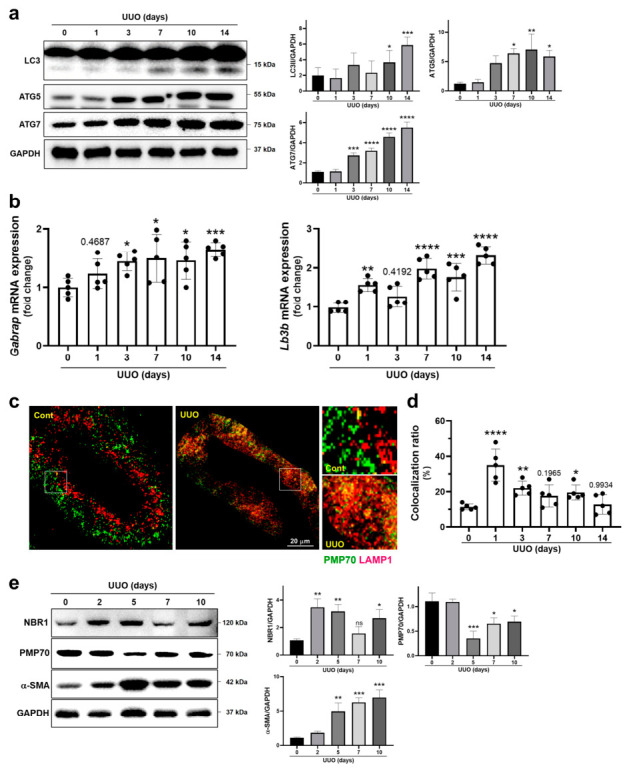
Autophagy in peroxisomes observed in UUO models. UUO was performed in mice for indicated periods, and kidney tissue was harvested. (**a**) Western blot and quantification of LC3, ATG5, and ATG7 antibodies. GAPDH was used as a loading control. * *p* < 0.05, ** *p* < 0.01, *** *p* < 0.001, **** *p* < 0.0001, compared with the control group. (**b**) Relative mRNA expression level of *Gabarap* and *Lc3b*. Data are expressed as mean ± SD (*n* = 5 individual mice per group). Statistical analysis was performed via one-way ANOVA followed by Tukey’s post hoc test (*p* = 0.4647 for control vs. 1 d and *p* = 0.4192 for control vs. 3 d; * *p* < 0.05, ** *p* < 0.01, *** *p* < 0.001, and **** *p* < 0.0001, compared with the control group). (**c**,**d**) Immunofluorescence staining and quantification histogram of PMP70 and LAMP1, scale bar = 20 mm. Data are expressed as mean ± SD (*n* = 5 mice per experimental group). Statistical analysis was performed via one-way ANOVA followed by Tukey’s post hoc test (*p* = 0.1965 for control vs. 7 d and *p* = 0.9934 for control vs. 14 d; * *p* < 0.05, ** *p* < 0.01, and **** *p* < 0.0001, compared with the control group). (**e**) Western blot and quantification of NBR1, PMP70 and α-SMA antibodies. GAPDH was used as a loading control. Data are expressed as mean ± SD (*n* = 9 individual mice per group). Statistical analysis was performed via one-way ANOVA followed by Tukey’s post hoc test (* *p* < 0.05, ** *p* < 0.01, *** *p* < 0.001, compared with the control group; ns, not significant).

UUO induces autophagy in kidney cells, particularly in the proximal tubules and interstitial cells [28]. Next, to explore the possibility that a decrease in the number of peroxisomes is involved in their degradation, we examined the autophagy process. The expression of autophagy markers, including LC3B, ATG5, and ATG7, was increased after UUO (Figure 3a), and the mRNA levels of *Gabarap* and *Lc3b* were also escalated in a time-dependent manner (Figure 3b). We also performed IF to detect the co-localization of LAMP1 and PMP70, an indicator of lysosome–peroxisome fusion that occurs during the pexophagy process. As shown in Figure 3c, co-localization of PMP70 and LAMP1 was markedly increased 1 day after UUO. Additionally, the ratio of co-localization gradually decreased, returning to the same level as that in the control, which might be due to the elimination of pre-existing peroxisomes (Figure 3d).

Next, to determine whether the pexophagy receptor was involved in the progression of UUO, Western blot analysis was performed at 2, 5, 7, and 10 days after surgery. In the selective autophagic degradation of peroxisomes, receptors p62 and NBR1 cooperate to aggregate peroxisomes and link them to autophagosomes. While p62 expression is essential for inducing the initial aggregation of peroxisomes, NBR1 expression levels are a key factor determining peroxisome autophagic efficiency and function even in the absence of p62 [13]. As shown in Figure 3e, the expression level of NBR1 increased after UUO, which was accompanied by a decrease in PMP70 and an increase in α-SMA (Figure 3e). These data suggest that the decrease in peroxisomes in UUO is due to the autophagic degradation of peroxisomes.

### 3.4. UUO Is Mediated by Hypoxia and ROS

**Figure 4:**
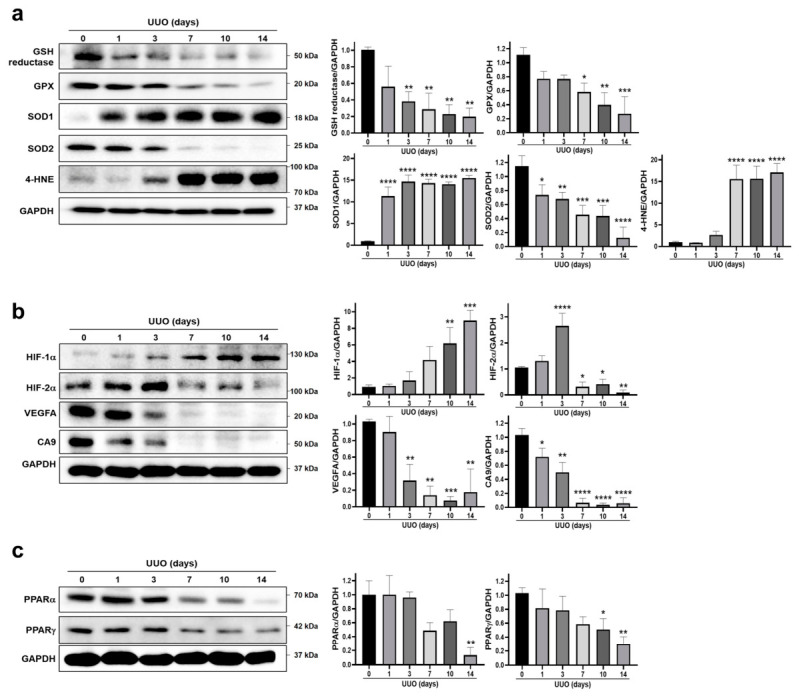
ROS and hypoxia are increased in UUO models. UUO was performed in mice for indicated periods, and kidney tissue was harvested. (**a**–**c**) Western blot and quantification of GSH reductase, GPX, SOD1, SOD2, 4-HNE, HIF-1a, HIF-2a, VEGFA, CA9, PPARa, and PPARg antibodies. GAPDH was used as a loading control. Data are expressed as mean ± SD (*n* = 9 individual mice per group). Statistical analysis was performed via one-way ANOVA followed by Tukey’s post hoc test (* *p* < 0.05, ** *p* < 0.01, *** *p* <0.001, **** *p* < 0.0001, compared with the control group).

UUO leads to reduced oxygen supply to renal tissue, creating a hypoxic environment that triggers the activation of the transcription factor HIF1, which regulates gene expression [29]. Therefore, we investigated the expression levels of ROS- and hypoxia-related genes. The expression of GSH reductase, GPX, and SOD2 was downregulated; however, the expression of SOD1 and 4-HNE was upregulated by UUO in a time-dependent manner (Figure 4a). Furthermore, HIF-1α expression increased, whereas that of HIF-2α initially increased and then decreased with UUO, accompanied by VEGFA and CA9 downregulation (Figure 4b).

Peroxisome activity and proliferation can be regulated by PPARs, which can also be activated by certain chemicals that interact with peroxides, suggesting a complex interaction between PPARs and peroxisomes [29]. In this study, PPARα and PPARγ expression decreased after UUO in a time-dependent manner (Figure 4c). Taken together, these results indicate that UUO may be mediated by imbalances in ROS status and hypoxic stress through PPAR signaling.

## 4. Discussion

In this study, we established an experimental mouse model of renal injury, and measured time-dependent changes in inflammation and fibrosis. To accurately separate the pathological effects of ligation from the systemic effects of surgery, anesthesia, and normal aging, it is ideal to include a temporally matched sham control at every time point being evaluated [30]. A limitation of this study is that, despite the collection of temporally matched sham control groups at each evaluation point, the analysis was primarily based on the baseline (day 0) control group. Although evidence that ureteral obstruction causes significant pathological changes in the contralateral non-obstructed kidney or alters baseline physiological markers over 14 days is limited, future studies utilizing fully temporally matched sham groups at all analysis stages would be useful for completely excluding the delayed systemic effects of surgical trauma and anesthesia.

We evaluated time-dependent systemic changes in BUN and serum creatinine levels alongside intrarenal inflammation and fibrosis. However, interpreting whole-body serum biomarkers in a unilateral UUO model requires utmost caution due to significant inherent limitations. Because the contralateral kidney remains completely intact, it rapidly undergoes compensatory hyperfiltration, which effectively masks the functional decline of the obstructed kidney and maintains systemic BUN and creatinine levels close to the physiological baseline [24]. Therefore, these serum parameters should not be overinterpreted as direct, isolated measures of obstructed-kidney function. While evaluating a combination of systemic serum data and unilateral urine biomarkers from each kidney could theoretically provide a more compartmentalized functional profile, our findings primarily rely on tissue-specific molecular alterations to accurately reflect the progressive unilateral injury and subsequent redox imbalance induced by UUO.

In the present study, we observed a distinct antioxidant response characteristically marked by an increase in SOD1 and 4-HNE levels alongside a concomitant decrease in SOD2 expression within the fibrotic kidneys of UUO mice, pointing to compartmentalized oxidative stress. In contrast, Fan et al. reported an alternative phenotype where SOD2 expression remained largely unchanged, suggesting that both SOD1 and SOD2 were generally downregulated in their specific experimental setting, which subsequently drove oxidative stress and cell apoptosis [31]. This discrepancy between our findings and the report by Fan et al. may stem from subtle variations in model kinetics or tissue harvesting timelines, underscoring the complex, context-dependent regulation of specific SOD isoforms during chronic renal injury.

Typically, autophagy is induced as a protective mechanism against this hypoxic and oxidative stress; however, autophagy dysfunction exacerbates fibrosis and renal tubule damage. Autophagy dysfunction and increased apoptosis are presumed to be the major mechanisms involved in increased renal fibrosis under UUO [32]. The autophagy process relies on autophagy-related (ATG) proteins. Both ATG5 and ATG7 are key components in the formation of autophagosomes, structures that engulf cellular material destined for degradation [33]. The data obtained in this study show that in the UUO mouse model, the expression of GSH reductase, GPX, and SOD2 decreases in a time-dependent manner, while the expression of SOD1 and 4-HNE increases. Additionally, during UUO, it was observed that the expression of autophagy markers such as LC3B, ATG5, and ATG7, as well as the specific pexophagy cargo receptor NBR1, markedly increased, while the expression of the peroxisomal membrane protein PMP70 decreased. This reciprocal expression profile is characteristically mediated by the active lysosome–peroxisome fusion that occurs during selective peroxisomal autophagy. Taken together, these findings strongly suggest that the progressive loss of peroxisomes observed during UUO is directly driven by the accelerated autophagic degradation of peroxisomes triggered by the severe intrarenal redox imbalance.

PPARα plays a pivotal role in regulating autophagy and fatty acid metabolism [4]. In addition, it is highly expressed in the proximal tubules and is involved in maintaining energy balance and regulating lipid metabolism in the kidney. Fibrates, which are a type of PPARα agonist, have shown protective effects on kidney function in conditions such as dyslipidemia and diabetic nephropathy [34]. Additionally, all three PPAR isoforms (α, β/δ, and γ) are expressed in the kidney and are associated with various renal pathophysiological conditions [29]. Peroxisomes respond to stimuli acting through PPAR, which increases peroxisome metabolism and proliferation. The potential protective effects of peroxisomal ROS modulators in renal fibrosis have been studied [1]. PPARγ can also regulate autophagy [4]. Consistent with this, PPARα and PPARγ expression was decreased after UUO in this study. However, the relation between autophagic degradation of peroxisomes and peroxisomal biogenesis through PPAR activation still requires further study.

We demonstrated that UUO increased ROS production through peroxisomal degradation and dysregulation of mitochondrial antioxidant enzymes. Oxidative stress, caused by increased levels of endogenous and exogenous ROS, is a major cause of organ fibrosis, a common pathway leading to organ dysfunction and death [35]. Renal dysfunction induces oxidative stress, accelerating the progression of kidney diseases such as AKI, CKD, and nephrotic syndrome [36]. ACOX1 is the primary and rate-limiting enzyme responsible for the initial step of VLCFA degradation [37]. Our results suggest that decreased ACOX1, DBP, and catalase with UUO may affect lipid metabolism through oxidative stress.

Although a large body of evidence formed over the past several decades has identified many essential pathogenic mechanisms that promote fibrosis in hypoxia, the precise molecular mechanisms involved and the interplay between the pathways involved are still not fully understood [29,38]. HIFs are the main transcription factors activated in response to hypoxic stress [38,39]. In this study, although direct HIF transcriptional reporter activity was not specifically measured, an apparent reduction in downstream HIF-2α signaling was inferred through the significantly decreased expression levels of its canonical target genes, such as VEGFA and CA9. Intriguingly, these target molecules are tightly linked to microenvironmental remodeling; VEGF actively modulates vascular permeability and angiogenic responses under ischemic conditions, while CA9 serves as a critical pH regulator characteristically upregulated in response to tissue hypoxia [40]. Collectively, our findings tentatively suggest that the downstream regulatory capacity of HIF-2α over VEGF and CA9 expression might be compromised, potentially secondary to the severe disruption of oxygen homeostasis induced by pexophagy [41]. However, further functional assays, such as luciferase reporter or chromatin immunoprecipitation (ChIP) analyses, are required to definitively corroborate whether this altered expression is directly mediated by a decline in HIF-2α transcriptional activity.

Pexophagy can be induced by various stress conditions or peroxisomal dysfunction. Autophagy adaptor proteins, such as NBR1 and p62, interact with PMP70 and other peroxisomal membrane proteins (PMPs) to recruit target peroxisomes into autophagosomes [3,42]. To resolve oxidative stress in damaged peroxisomes, ubiquitinated PEX5 interacts with the autophagy receptor p62, thereby recruiting the autophagy–lysosomal machinery for their removal [34,43]. In addition, specific proteins involved in this process, including PEX3, PEX13, and PEX14, are targeted by autophagy receptors such as p62 or NBR [3,44,45]. Furthermore, PMP70 is ubiquitinated in response to cellular stress; this ubiquitinated PMP70 is subsequently recognized by autophagy receptor NBR1, which promotes the targeting of peroxisomes for degradation via pexophagy [13,44]. While the mechanisms and functions of pexophagy have been thoroughly studied and extensively reviewed, there are still significant gaps in our knowledge regarding the complex regulatory network involving these adaptors. In exploring the molecular mechanism of pexophagy during UUO, the selection of experimental time points was strategically tailored to the distinct kinetics of the respective downstream pathways. While macro-level pathological alterations and tissue fibrosis characteristically mature over 14 days (evaluated at days 1, 3, 7, 10, and 14), cellular autophagic clearance mechanisms—specifically those mediated via the cargo receptor NBR1—operate on a highly dynamic and transient timeline. Because NBR1-mediated cargo targeting typically spikes during the acute-to-intermediate phase of cellular stress before undergoing autophagosomal degradation, evaluating NBR1 expression at days 2, 5, 7, and 10 allowed us to systematically capture the active window of pexophagy induction. This kinetic divergence accounts for the adjusted time points utilized for the NBR1 assays, ensuring that transient signaling events were not overlooked during the prolonged 14-day fibrotic progression. Nevertheless, a critical limitation of the present study is that the observed links between progressive peroxisome loss, elevated NBR1 expression, and metabolic dysfunction remain fundamentally correlative. Since direct genetic or pharmacological interventions targeting the pexophagic machinery were not implemented in this experimental design, this process must not be presented as a proven causal driver of renal fibrosis. Instead, accelerated peroxisomal degradation should currently be interpreted as a prominent pathological feature tightly synchronized with UUO-induced tissue involution. Future studies utilizing cell-type-specific conditional knockout models are strictly warranted to definitively untangle this causal architecture and map out the functional dynamics of pexophagy flux during CKD.

The de novo synthesis of peroxisomes requires peroxisomal biogenesis factors called peroxins. Among them, PEX3, PEX16, and PEX19 are essential for the import and insertion of PMP [46,47]. We speculated that UUO-induced fibrosis affects peroxisomal degradation pathways, as evidenced by the targeted decline in PMP70 and PEX14, rather than other organelle markers. However, despite a decrease in peroxisomal markers, the expression levels of proteins related to peroxisome biogenesis remained constant. While these observations may reflect a compensatory attempt to maintain peroxisome homeostasis, static expression levels alone cannot completely rule out defects in the functional biosynthetic mechanism [22]. A limitation of the current study is that peroxisomal biogenesis and degradation rates were inferred from steady-state protein levels; therefore, future flux analyses are required to completely delineate the relative contributions of impaired de novo biogenesis versus accelerated pexophagy in this model. Furthermore, it is worth noting that progressive unilateral injury induces profound tubular atrophy and epithelial remodeling, which could theoretically confound the interpretation of whole-kidney organelle dynamics. If the observed decline in peroxisomal components were merely a passive consequence of generalized proximal tubular cell loss, a proportional reduction in all cellular structures would be expected. However, our findings demonstrated that markers for the ER, Golgi apparatus, and mitochondria remained stable, implying a targeted, organelle-specific depletion of peroxisomes rather than a non-specific consequence of tissue involution. Nevertheless, while this multi-organelle control panel argues against passive cell loss as the sole driver, future studies utilizing compartment-specific localization—such as high-resolution co-staining with established proximal tubule markers like Lotus tetragonolobus lectin (LTL) or Aquaporin-1—are strictly warranted to definitively map out the independent degradation kinetics within specific nephron segments.

Both in vivo UUO and in vitro hypoxia models have exhibited the activation of HIF-1 and autophagy along with fibrotic changes [39]. Renal fibrosis was found to be reduced by the autophagy inhibitor chloroquine in a UUO model, and fibrotic changes were decreased by chloroquine and ATG7 depletion in renal proximal tubular cells. These results demonstrate a pro-fibrotic role of autophagy [48]. In addition, cisplatin-induced hypoxic renal injury in mice can be reversed by Sirt1 overexpression in proximal renal tubules, which upregulates catalase and reduces renal ROS production while preserving superoxide dismutase and function [49]. Autophagy impairment disrupts peroxisome dynamics, leading to an imbalance between ACOX, which generates H_2_O_2_, and malfunctional catalase, which in turn leads to a redox imbalance within the peroxisome [50]. Despite advances in our understanding of pexophagy’s role in renal disease, many reports still focus on macroautophagy. We therefore propose investigating organelle-specific autophagy mechanisms, particularly peroxisomal quality control in renal research [3].

## 5. Conclusions

**Figure 5:**
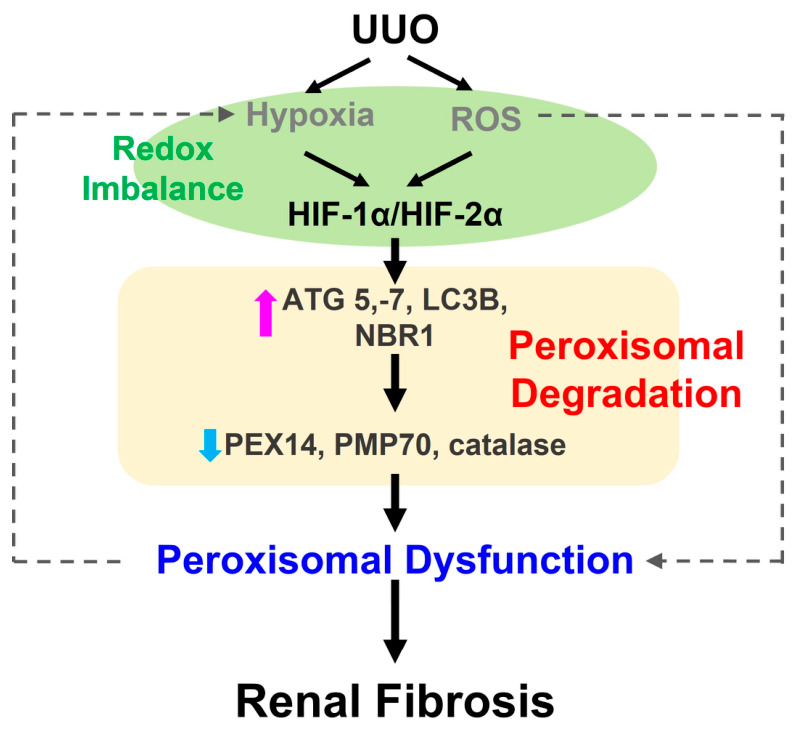
Summary of this study.

In conclusion, our study highlights that peroxisomal degradation is associated with the progression of chronic kidney injury in a UUO mouse model (Figure 5). While these synchronized alterations point toward a pathological correlation between compromised peroxisomal quality control and renal pathogenesis, whether this pathway functions as an active upstream driver or a secondary consequence of tissue damage remains to be definitively established in future interventional models.

## Data Availability

The original contributions presented in this study are included in the article/Supplementary Material. Further inquiries can be directed to the corresponding author.

## Supplementary Materials

The following supporting information can be downloaded at https://www.mdpi.com/article/10.3390/biology15130996/s1. Figure S1: Uncropped original images of Western blotting. Table S1: List of antibodies. Table S2: Primer sequences.

## Abbreviations

The following abbreviations are used in this manuscript:

UUO	Unilateral ureteral obstruction
FAO	Fatty acid oxidation
AKI	Acute kidney injury
CKD	Chronic kidney disease
BUN	Blood urea nitrogen
NGAL	Neutrophil gelatinase-associated lipocalin
GC-MS	Gas chromatography and mass spectrometry
KIM-1	Kidney injury molecule 1
ACOX1	Acyl-CoA oxidase 1
DBP	D-bifunctional protein
NBR1	Neighbor of BRCA1 Gene 1
