# Supplementary material for: Peroxisomal Degradation Correlates with the Progression of Kidney Injury in a UUO Mouse Model
Source: Biology (Basel). 2026 Jun 25;15(13):996. doi: 10.3390/biology15130996 (PMC13360078; doi:10.3390/biology15130996)
Supplement: Supplementary file 1 [file biology-15-00996-s001.zip › biology-4363317-Figure S1.pdf]

Figure 1

c

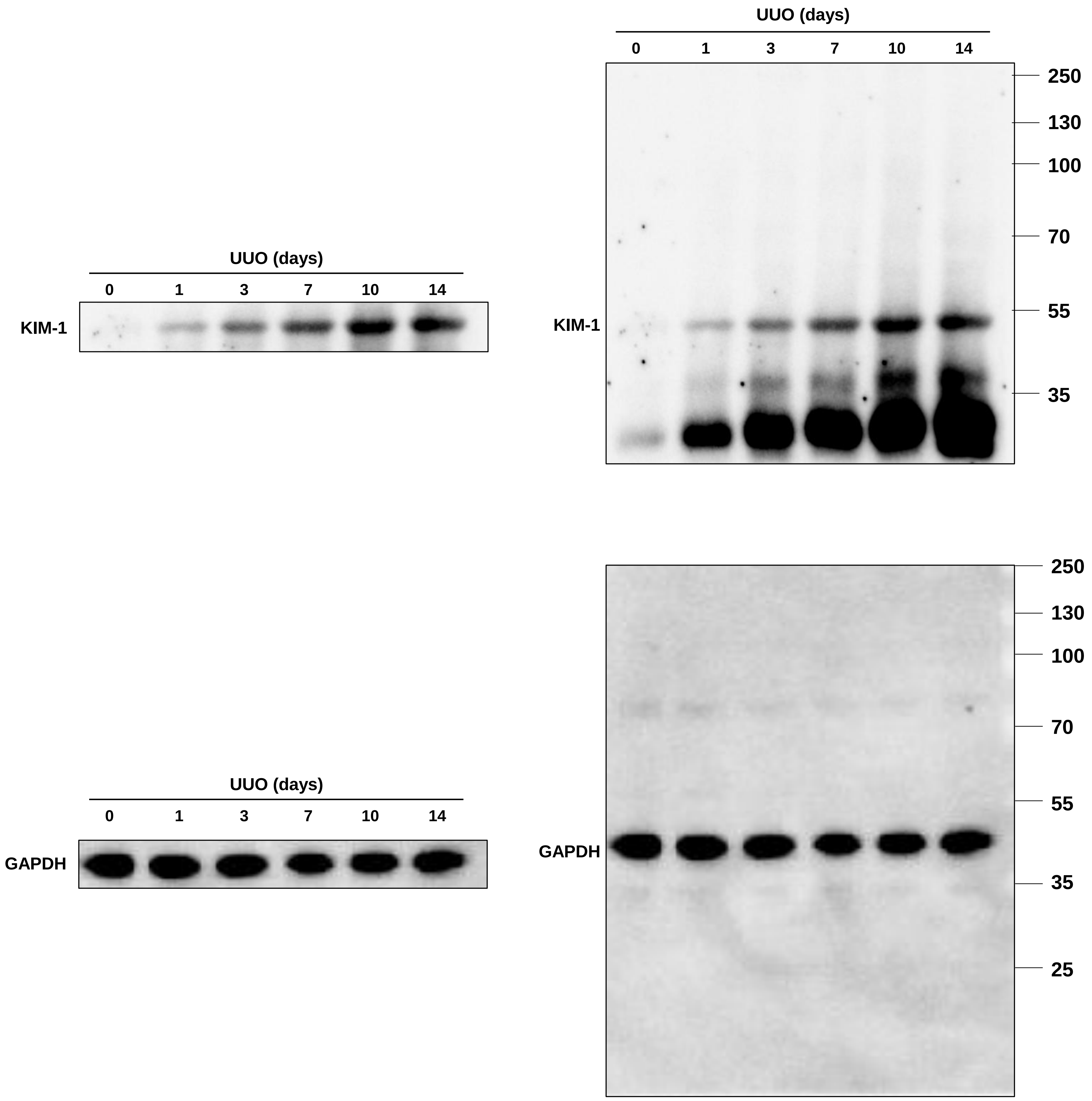
UUO (days)
0
1
3
7
10
14
KIM-1
UUO (days)
0
1
3
7
10
14
KIM-1
250
130
100
70
55
35
UUO (days)
0
1
3
7
10
14
GAPDH
GAPDH
250
130
100
70
55
35
25

Figure 1

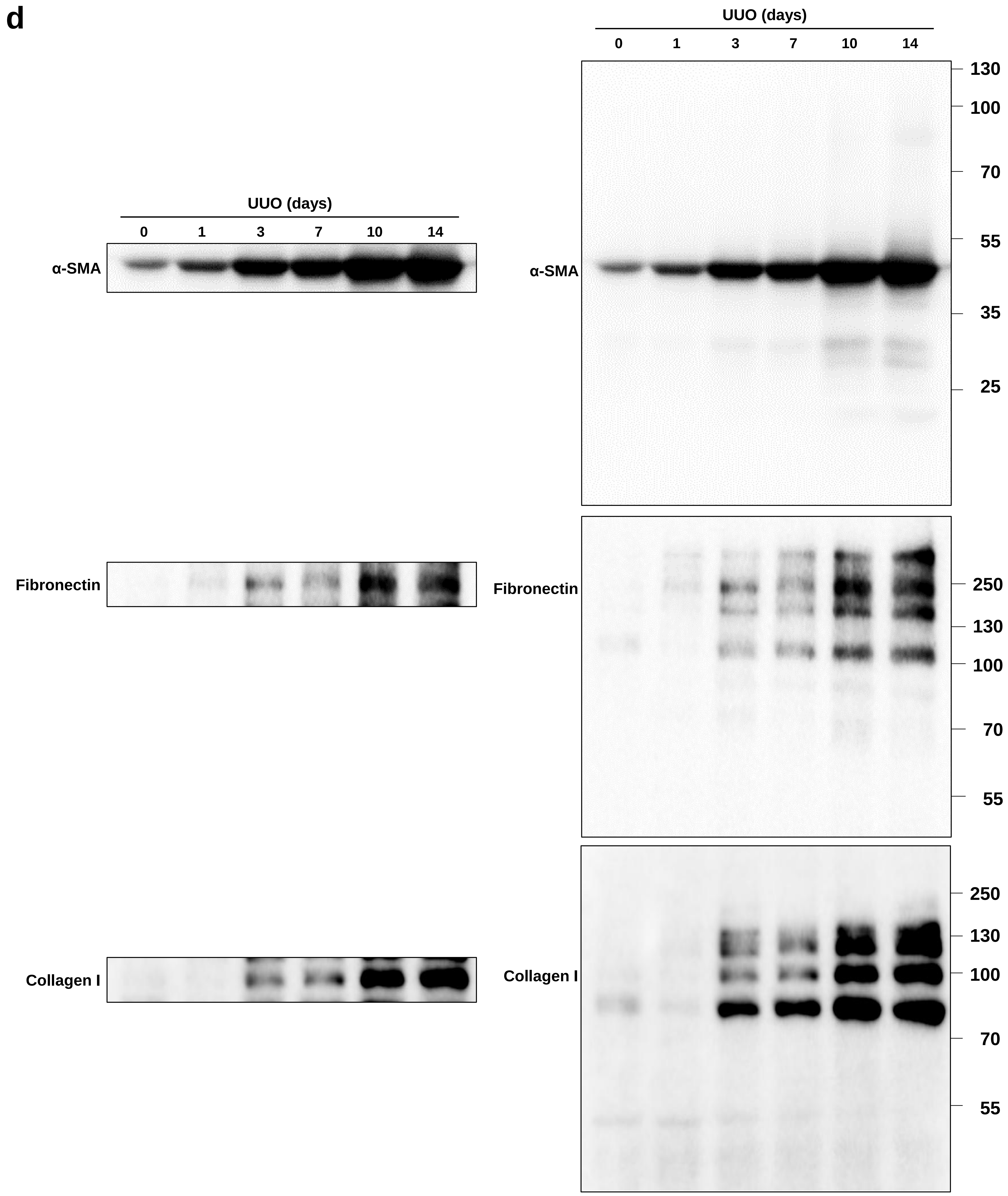
d
UUO (days)
0
1
3
7
10
14
α-SMA
Fibronectin
Collagen I
UUO (days)
0
1
3
7
10
14
130
100
70
55
35
25
α-SMA
Fibronectin
250
130
100
70
55
Collagen I
250
130
100
70
55

Figure 1

d

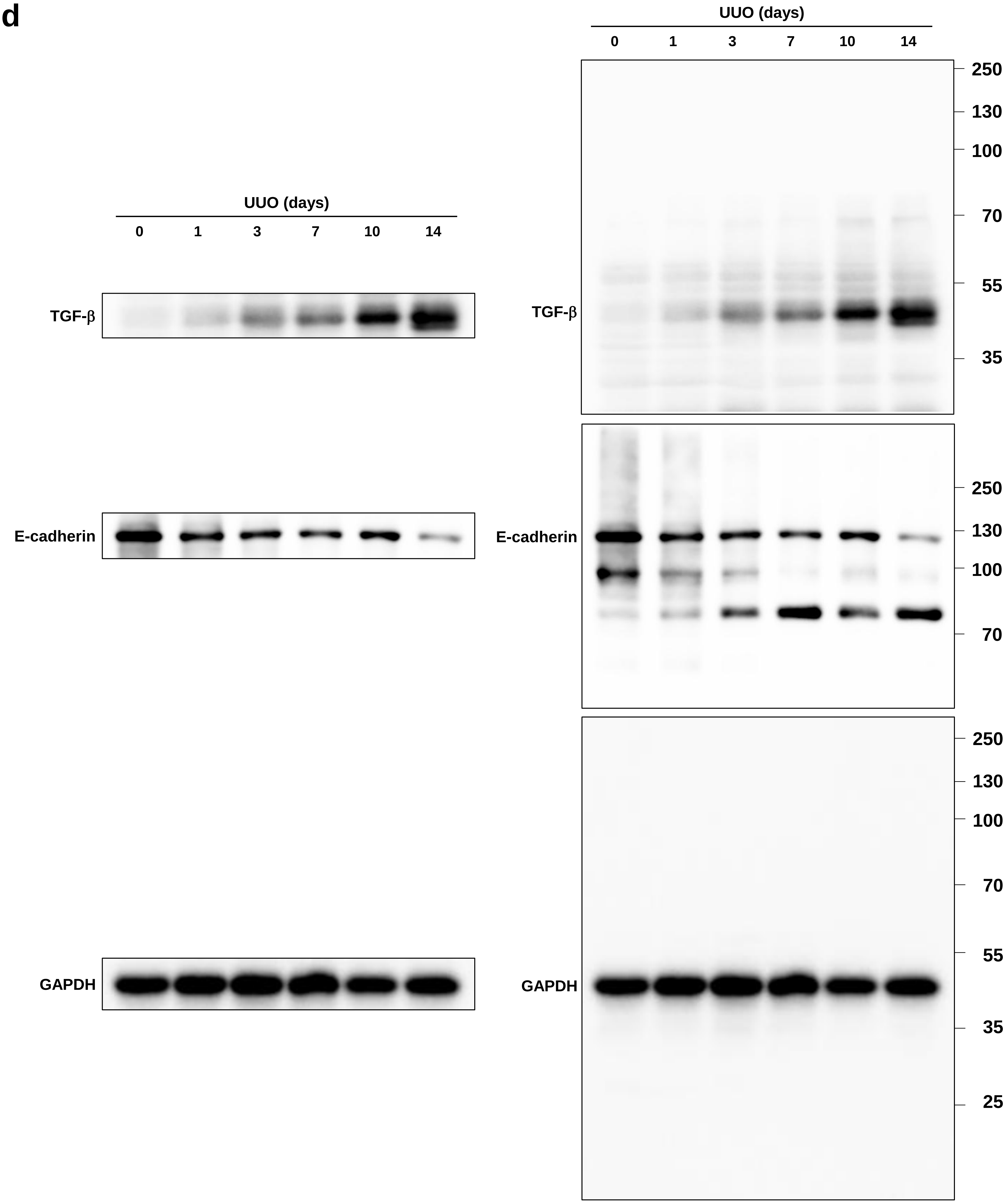
UUO (days)
0
1
3
7
10
14
TGF-β
E-cadherin
GAPDH
250
130
100
70
55
35
25

Figure 2

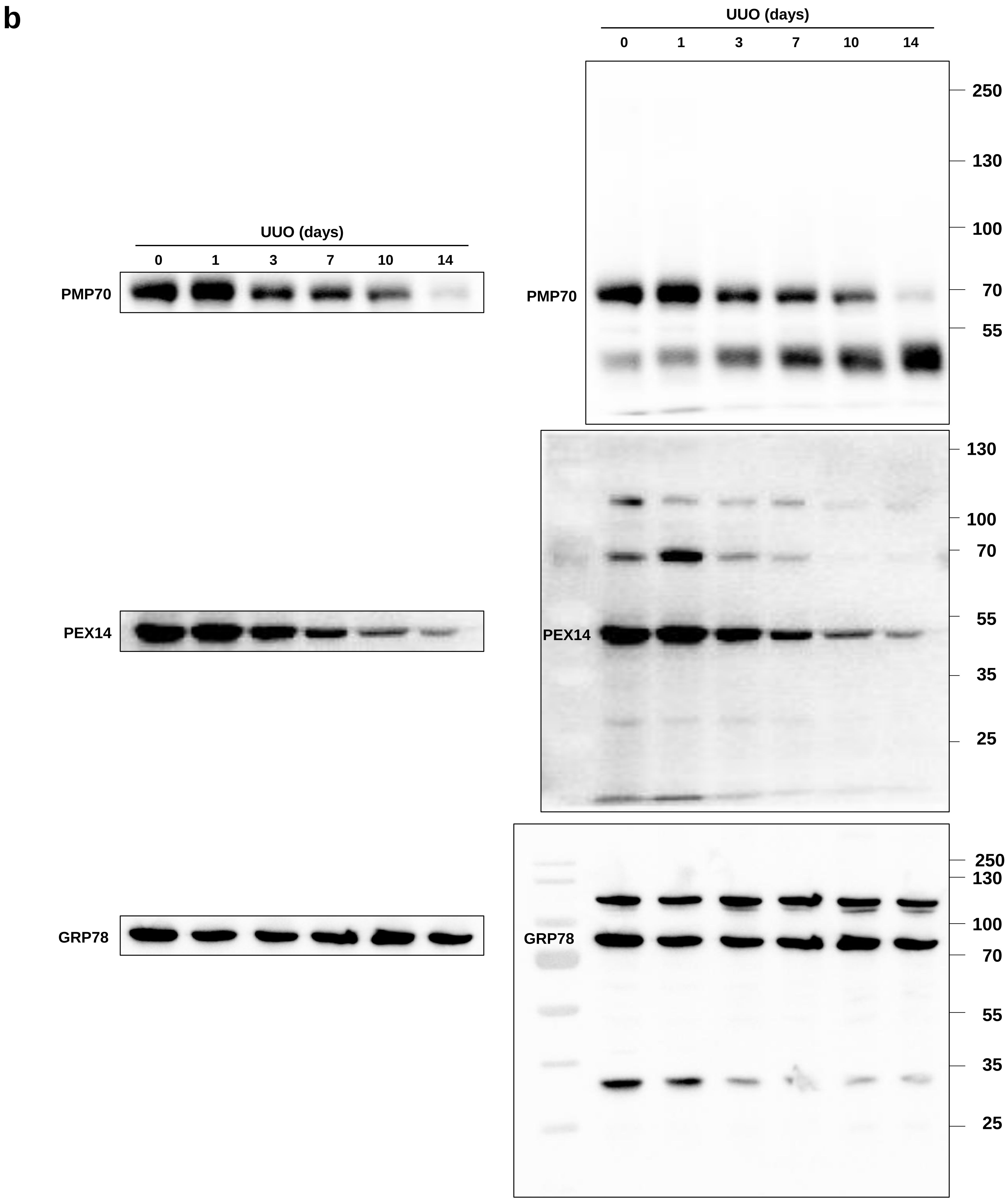
b
UUO (days)
0
1
3
7
10
14
PMP70
PEX14
GRP78
UUO (days)
0
1
3
7
10
14
PMP70
250
130
100
70
55
PEX14
130
100
70
55
35
25
GRP78
250
130
100
70
55
35
25

Figure 2

b

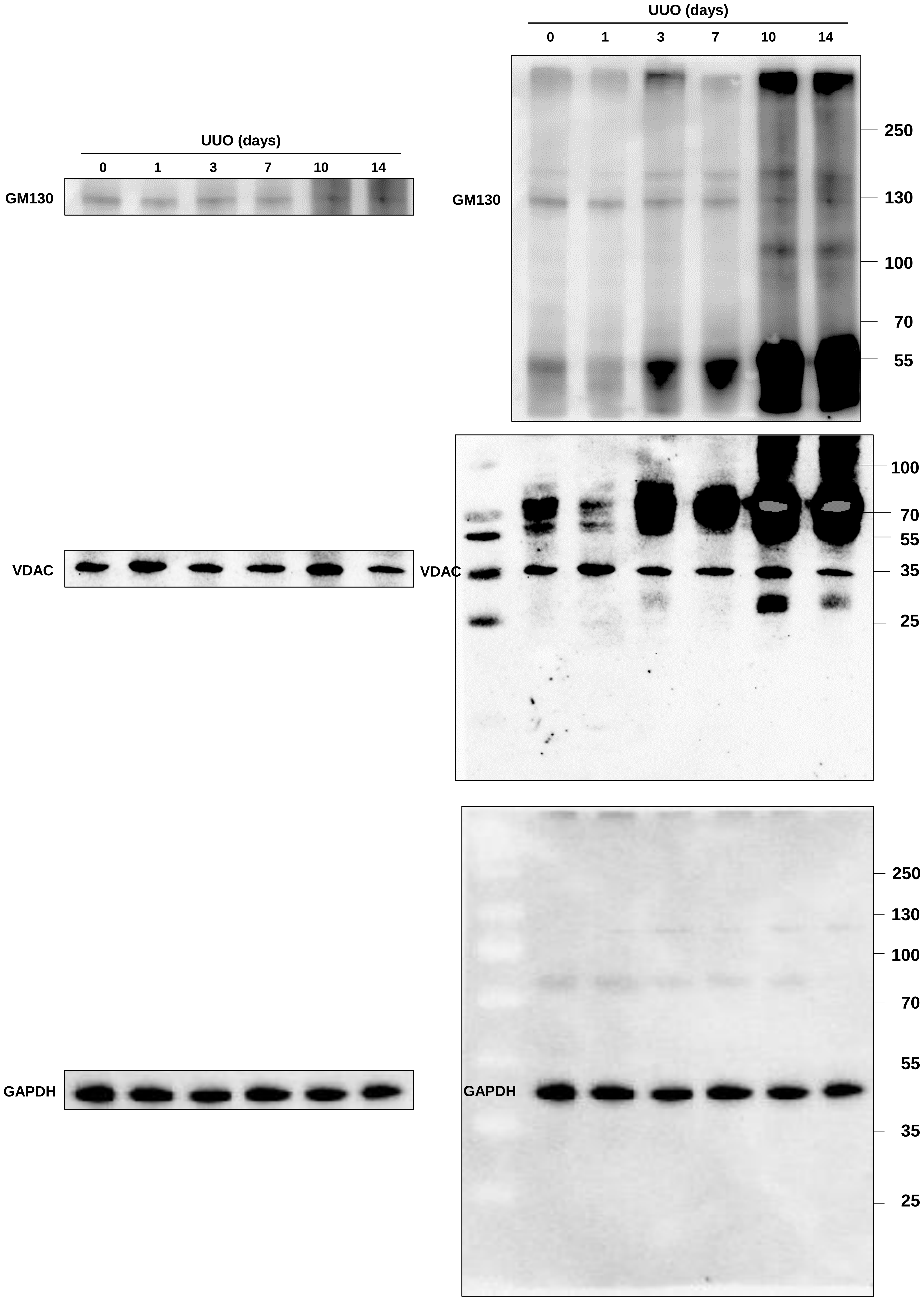
UUO (days)
0
1
3
7
10
14
GM130
VDAC
GAPDH
UUO (days)
0
1
3
7
10
14
GM130
250
130
100
70
55
VDAC
100
70
55
35
25
GAPDH
250
130
100
70
55
35
25

Figure 2

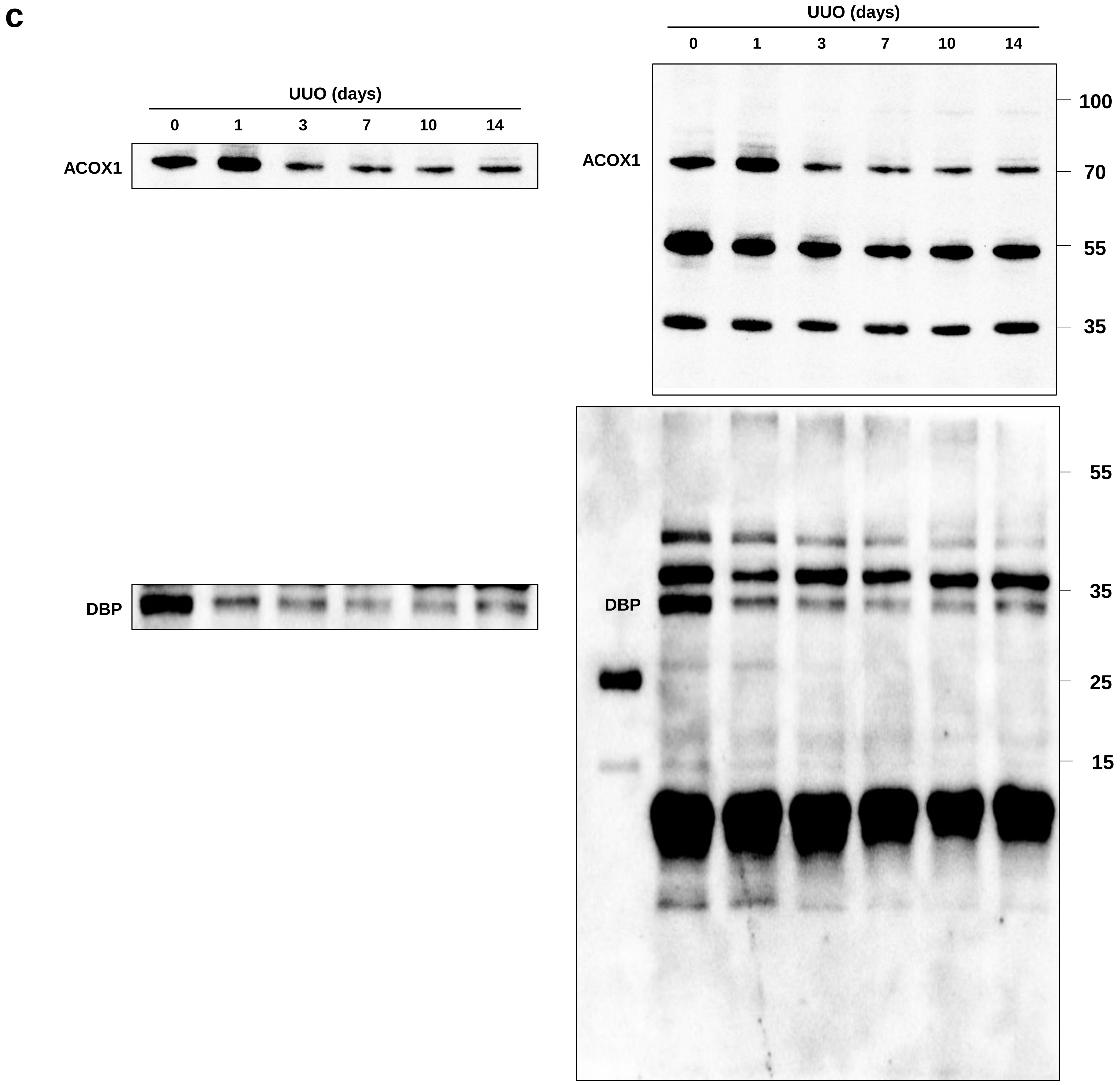

Figure 2

Figure 2

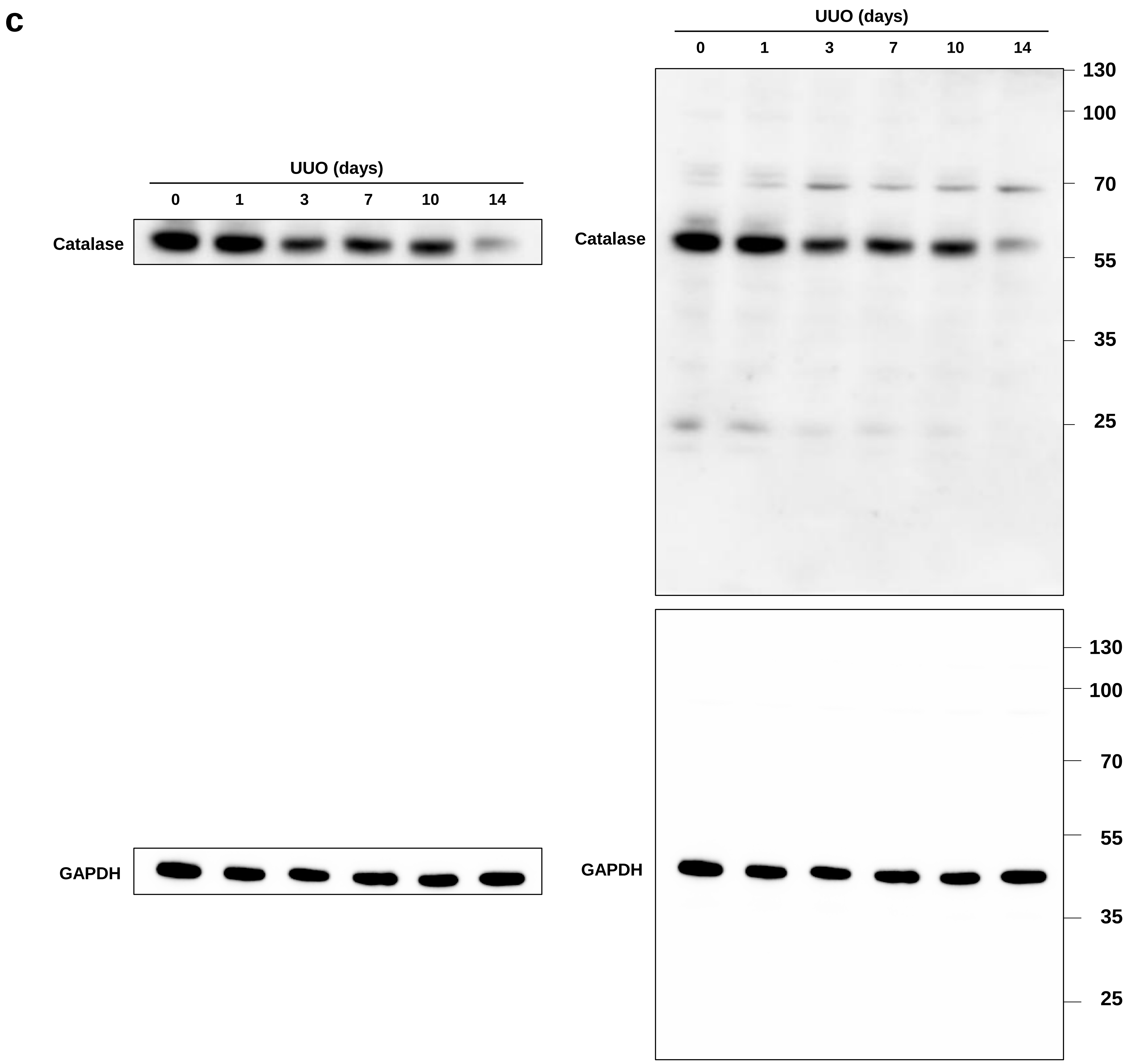
c
UUO (days)
0 1 3 7 10 14
Catalase
GAPDH
UUO (days)
0 1 3 7 10 14
Catalase
130
100
70
55
35
25
GAPDH
130
100
70
55
35
25

Figure 2

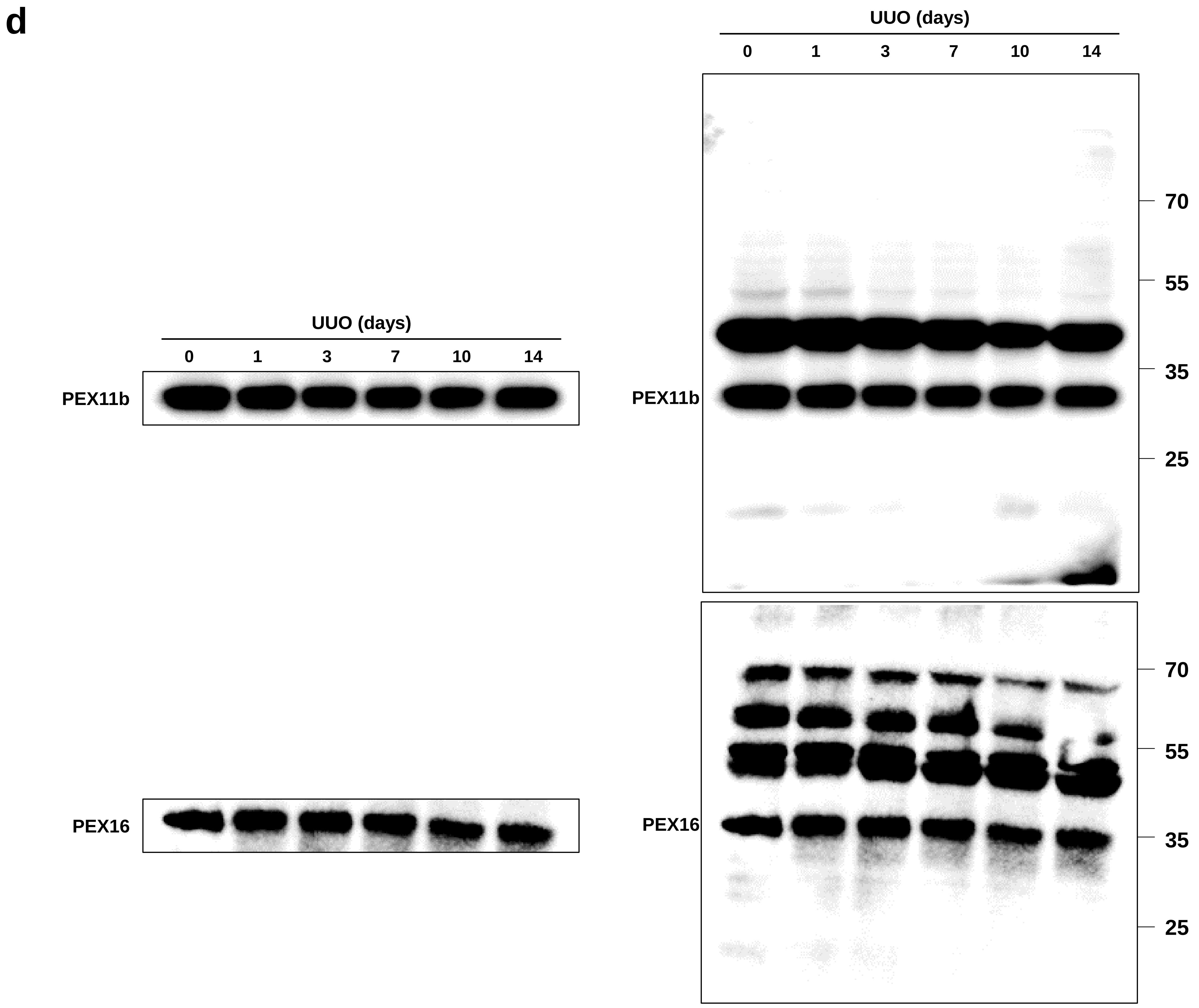

Figure 2

d

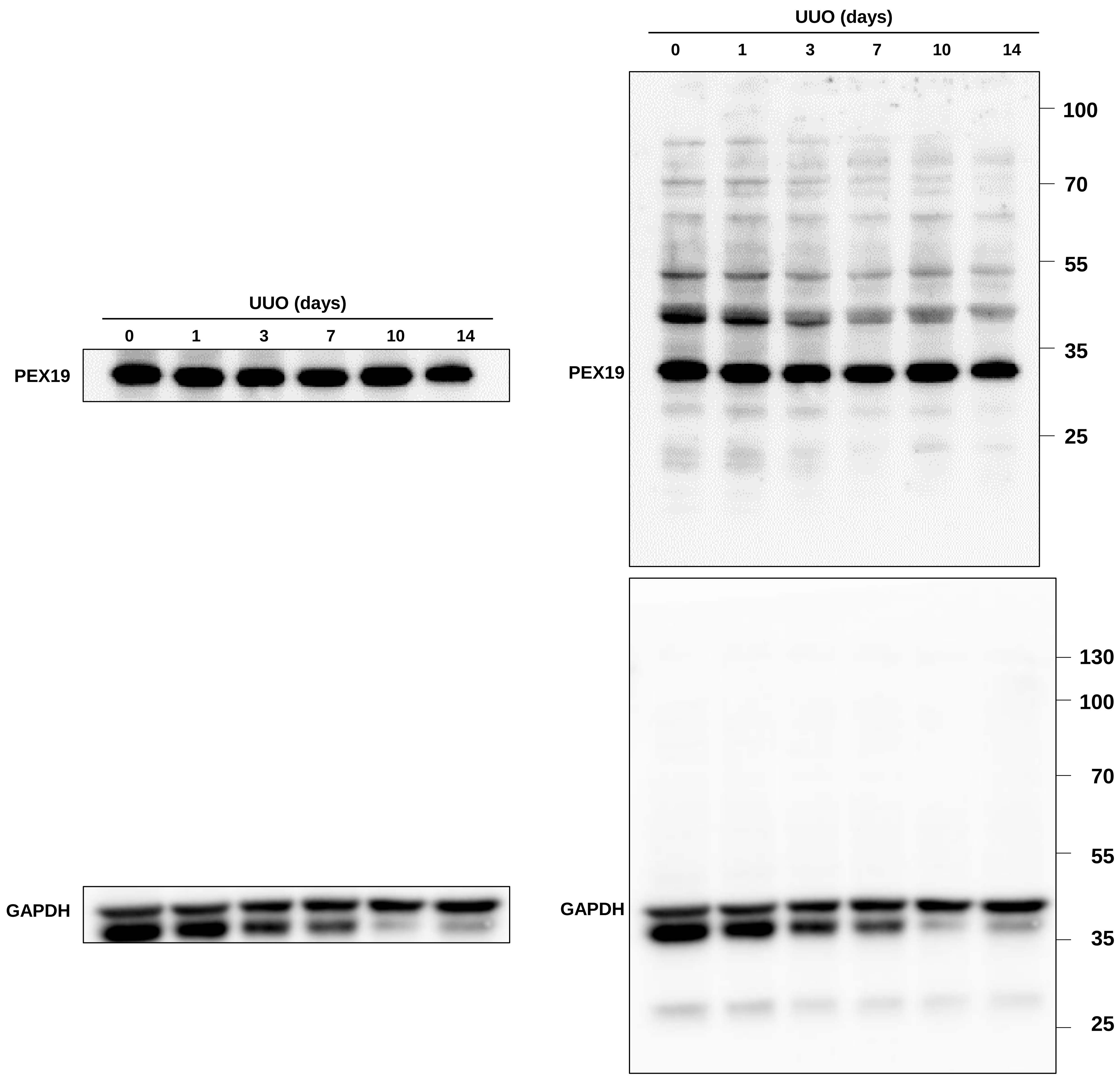
UUO (days)
0
1
3
7
10
14
PEX19
GAPDH
UUO (days)
0
1
3
7
10
14
PEX19
100
70
55
35
25
GAPDH
130
100
70
55
35
25

Figure 3

a

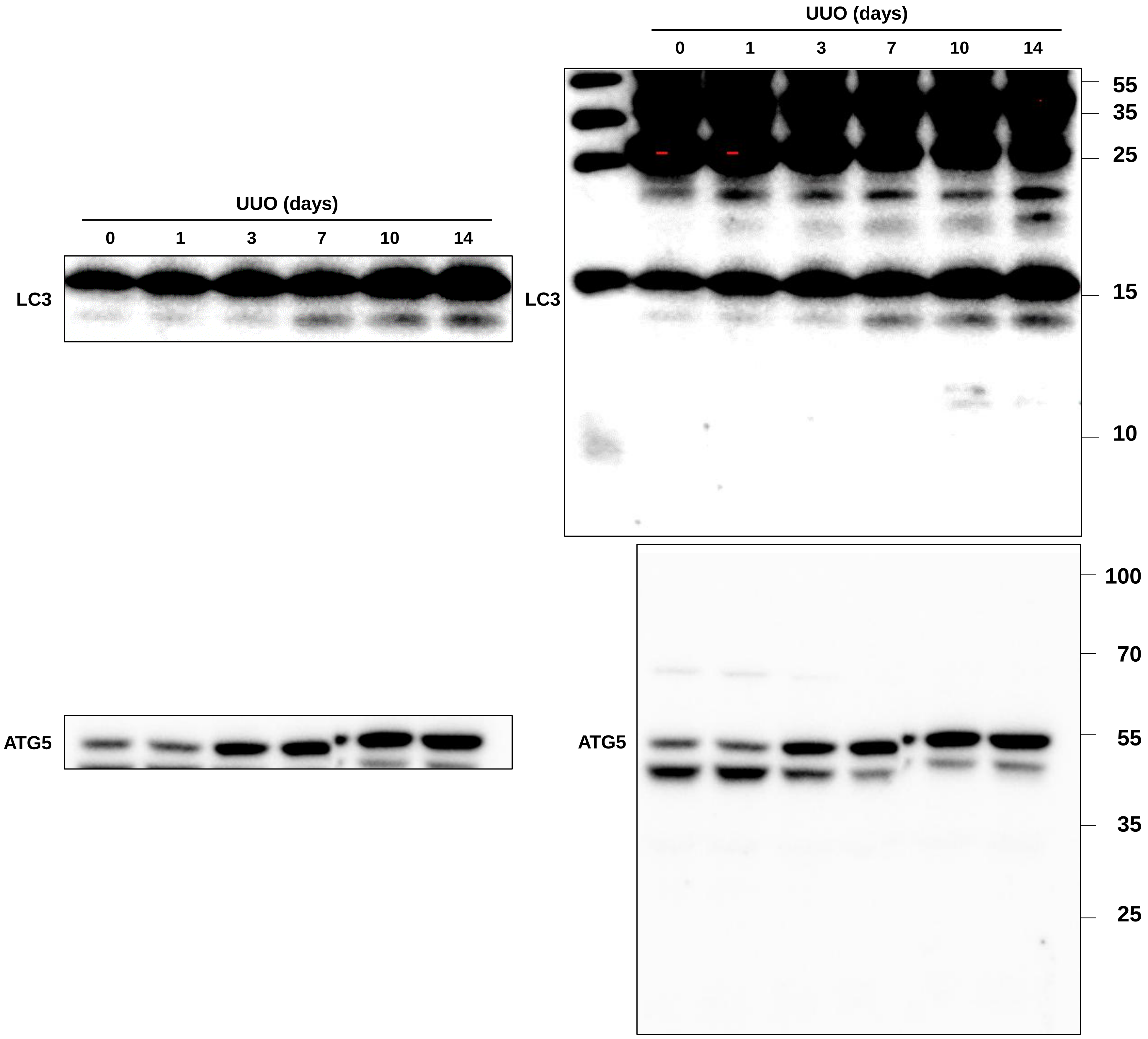
UUO (days)
0
1
3
7
10
14
LC3
ATG5
UUO (days)
0
1
3
7
10
14
LC3
55
35
25
15
10
ATG5
100
70
55
35
25

Figure 3

a

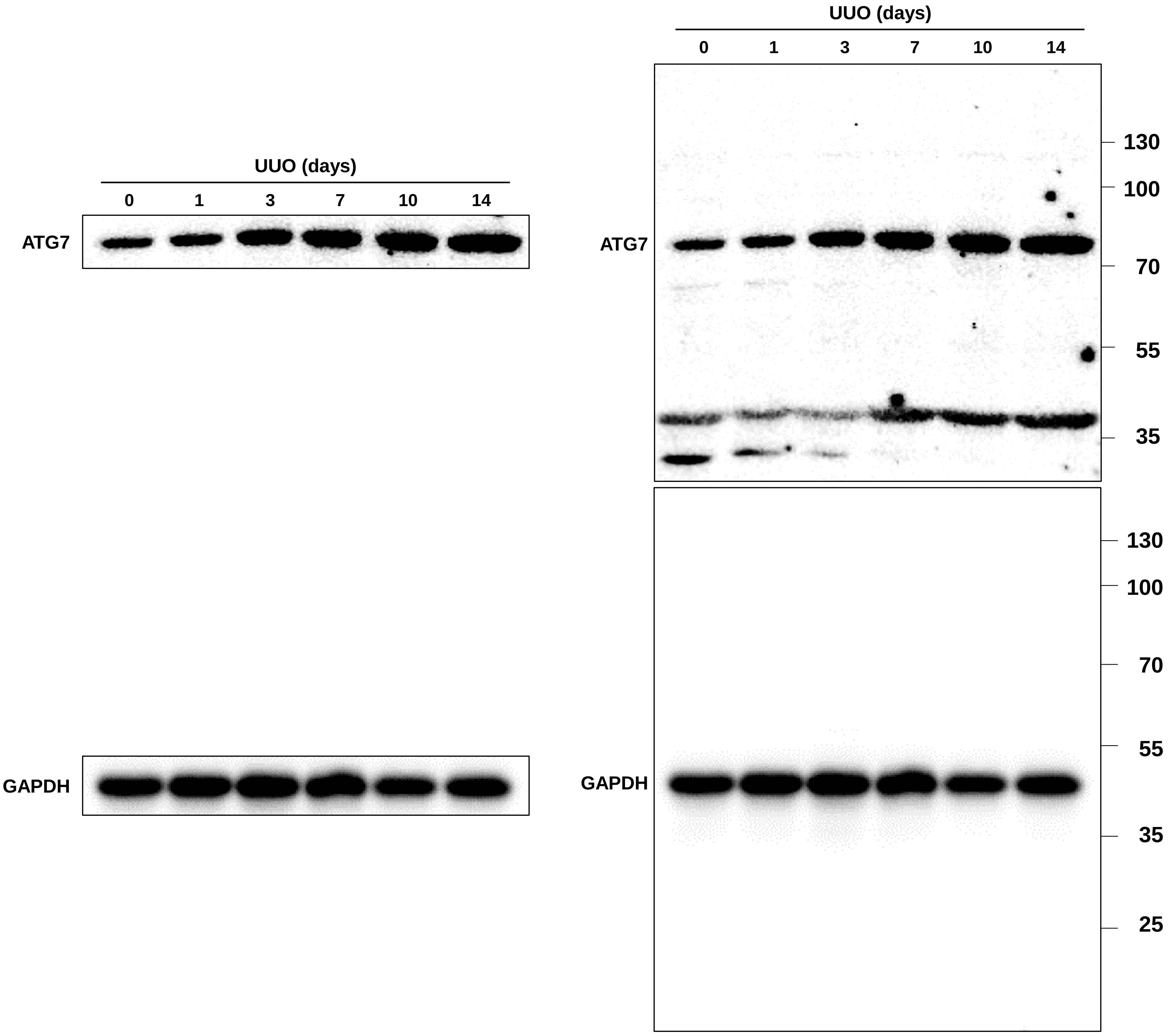
UUO (days)
0
1
3
7
10
14
ATG7
GAPDH
UUO (days)
0
1
3
7
10
14
ATG7
130
100
70
55
35
GAPDH
130
100
70
55
35
25

Figure 3

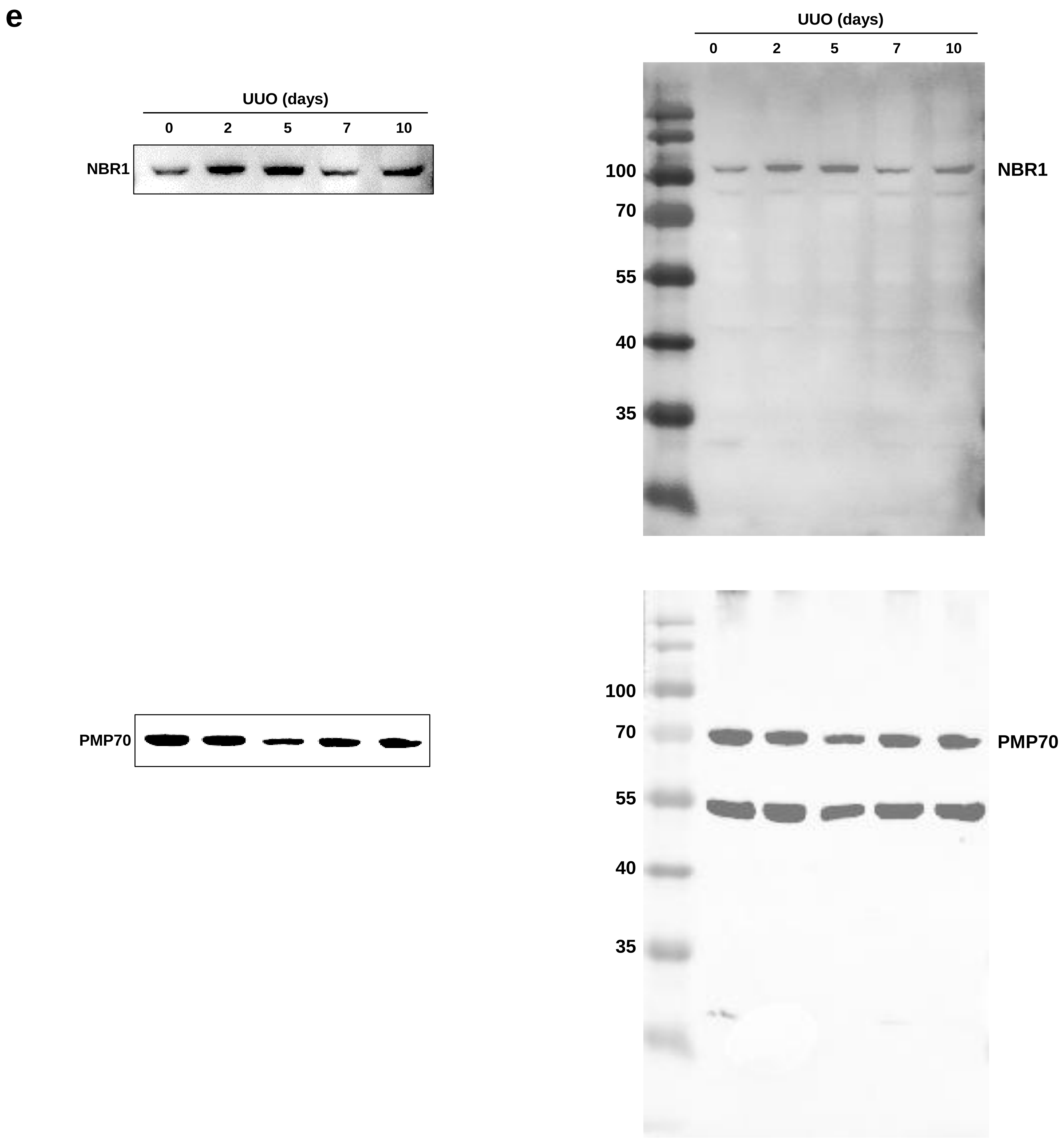
e
UUO (days)
0
2
5
7
10
NBR1
PMP70
UUO (days)
0
2
5
7
10
100
70
55
40
35
NBR1
100
70
55
40
35
PMP70

Figure 3

e

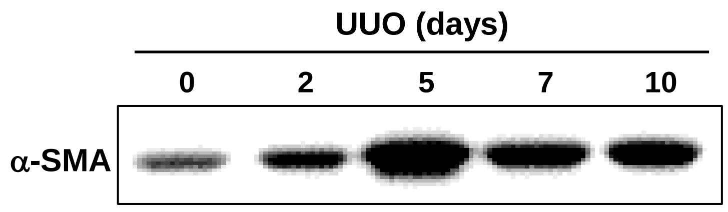

UUO (days)
0
2
5
7
10
α-SMA

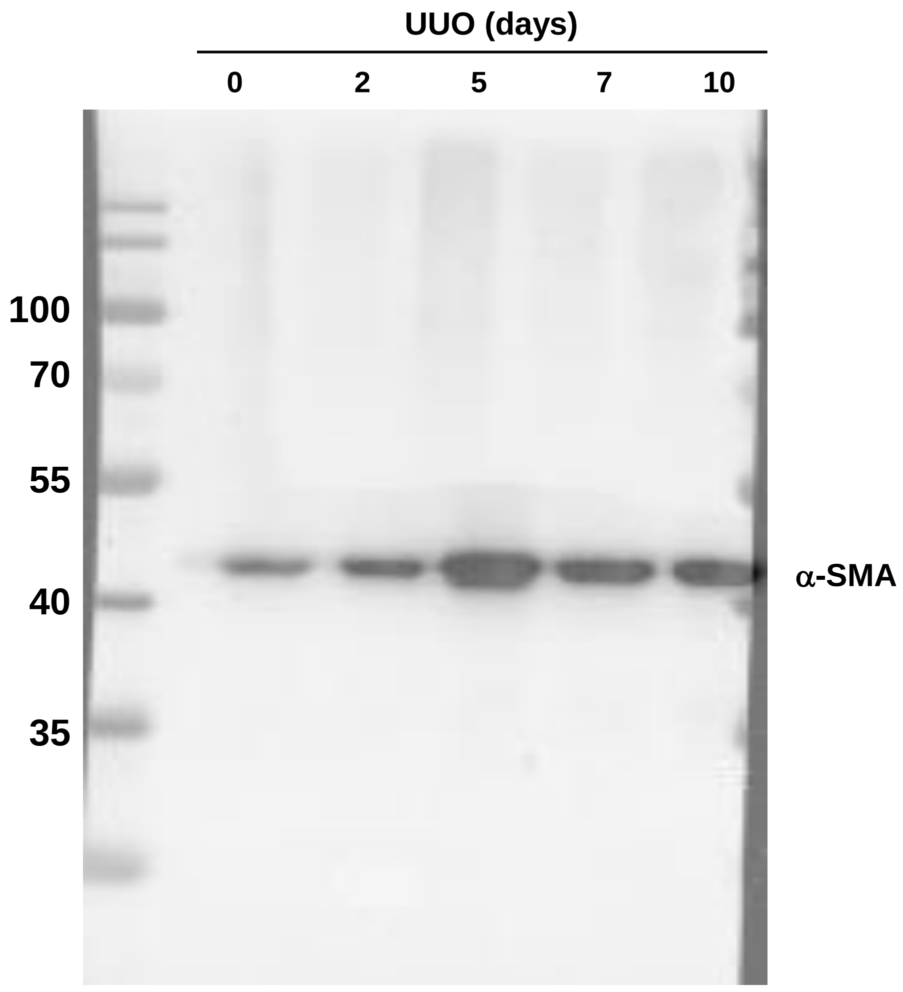

UUO (days)
0
2
5
7
10
100
70
55
40
35
α-SMA

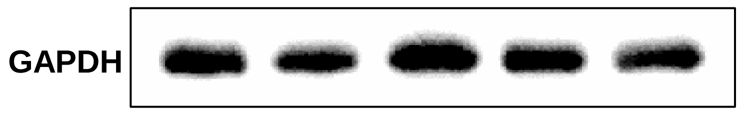

GAPDH

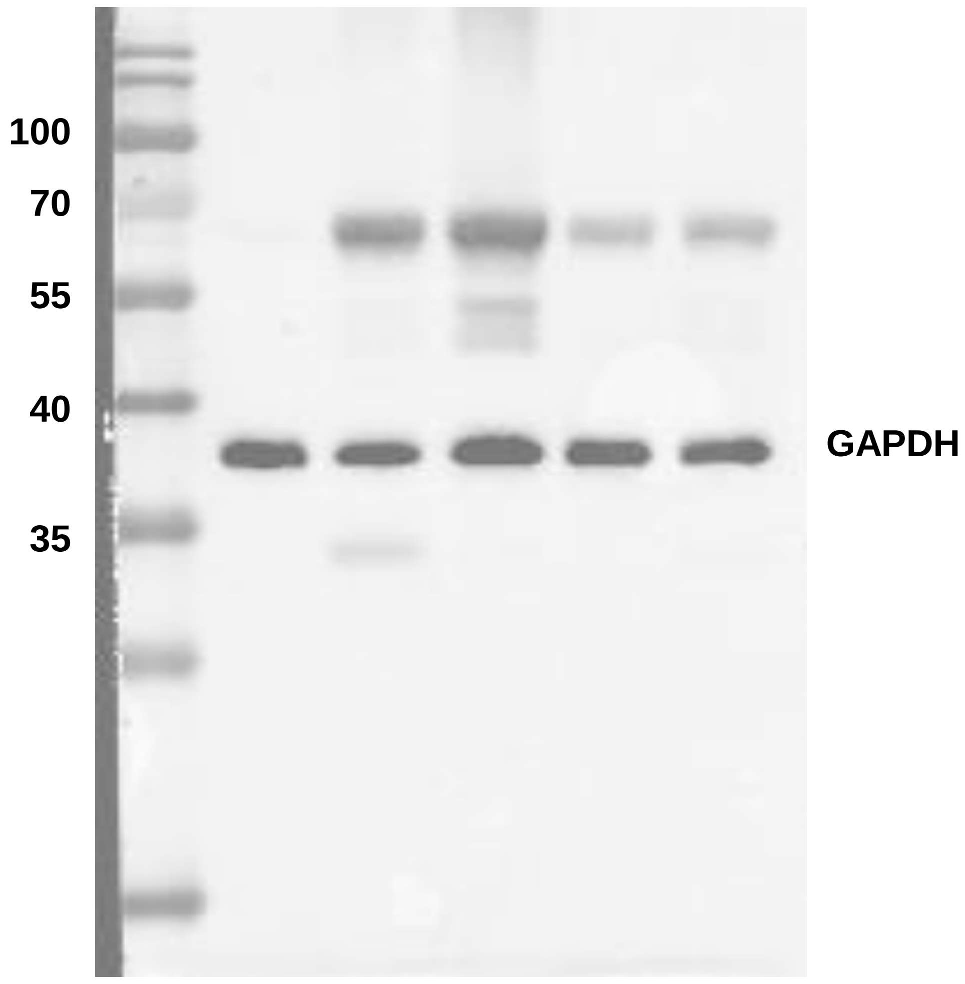

100
70
55
40
35
GAPDH

Figure 4

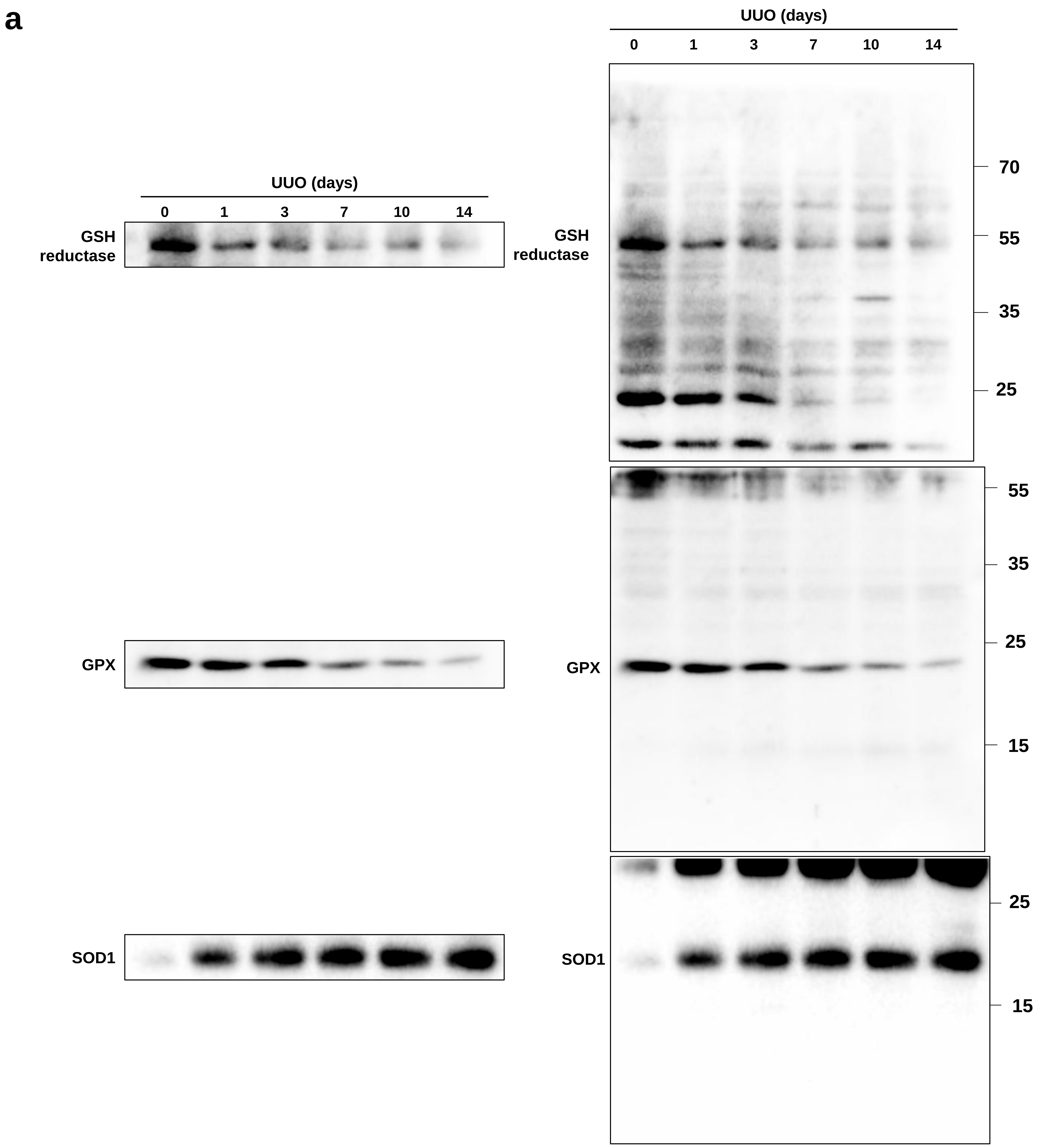
a
UUO (days)
0 1 3 7 10 14
GSH reductase
GPX
SOD1
UUO (days)
0 1 3 7 10 14
GSH reductase
70
55
35
25
GPX
55
35
25
15
SOD1
25
15

Figure 4

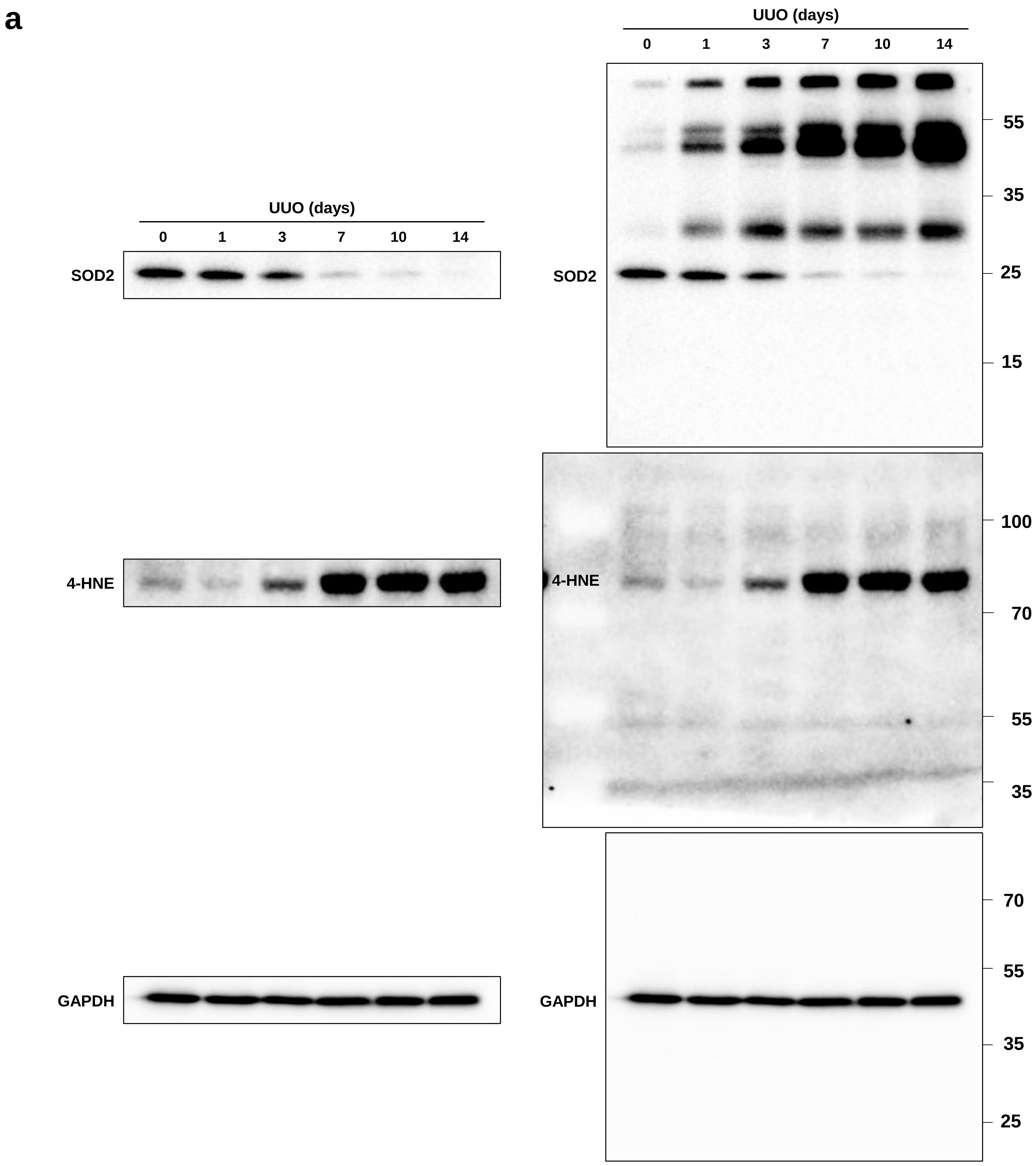
a
UUO (days)
0
1
3
7
10
14
SOD2
4-HNE
GAPDH
55
35
25
15
100
70
55
35
70
55
35
25

Figure 4

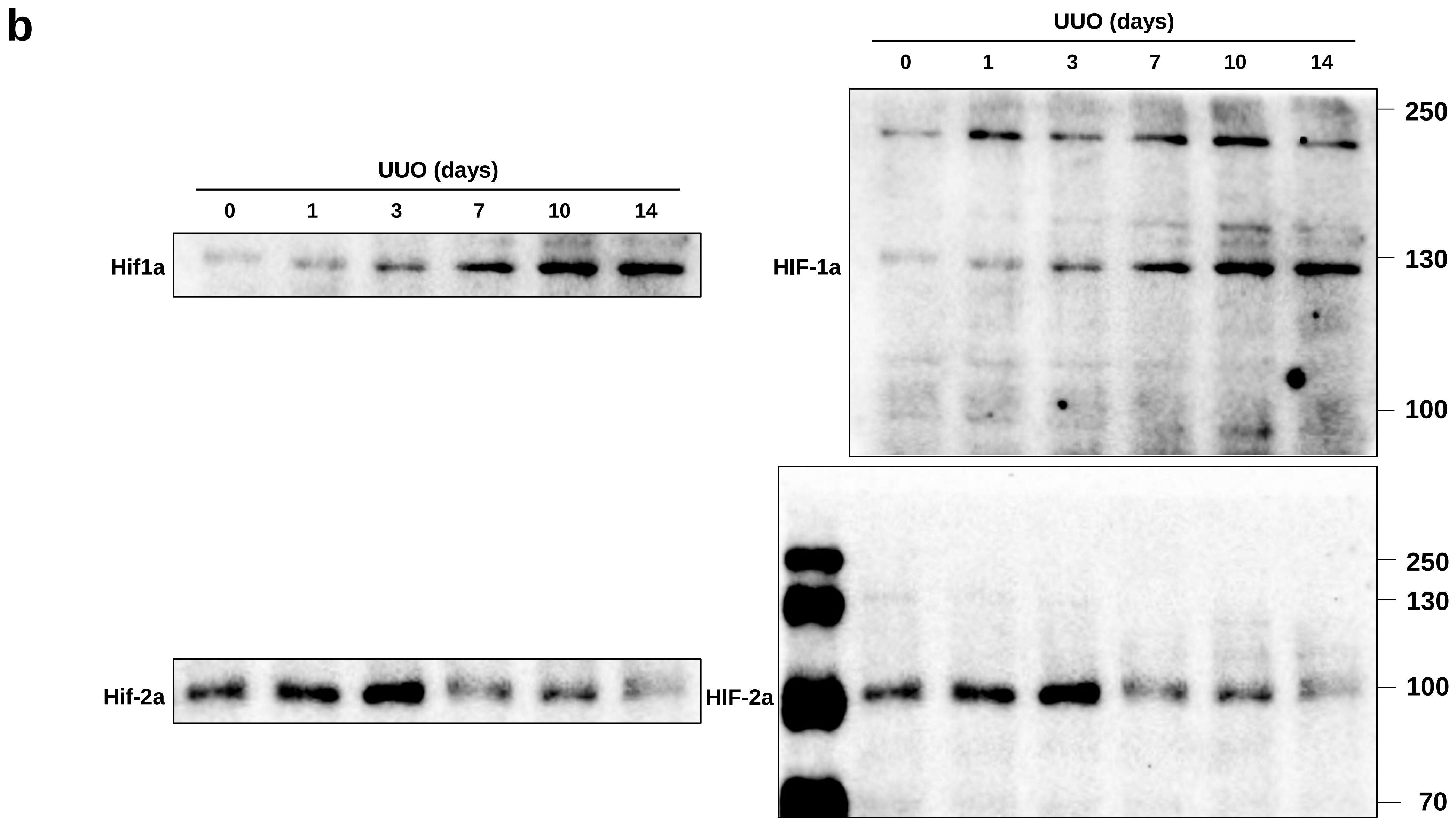

b
UUO (days)
0
1
3
7
10
14
Hif1a
Hif-2a
UUO (days)
0
1
3
7
10
14
HIF-1a
250
130
100
HIF-2a
250
130
100
70

Figure 4

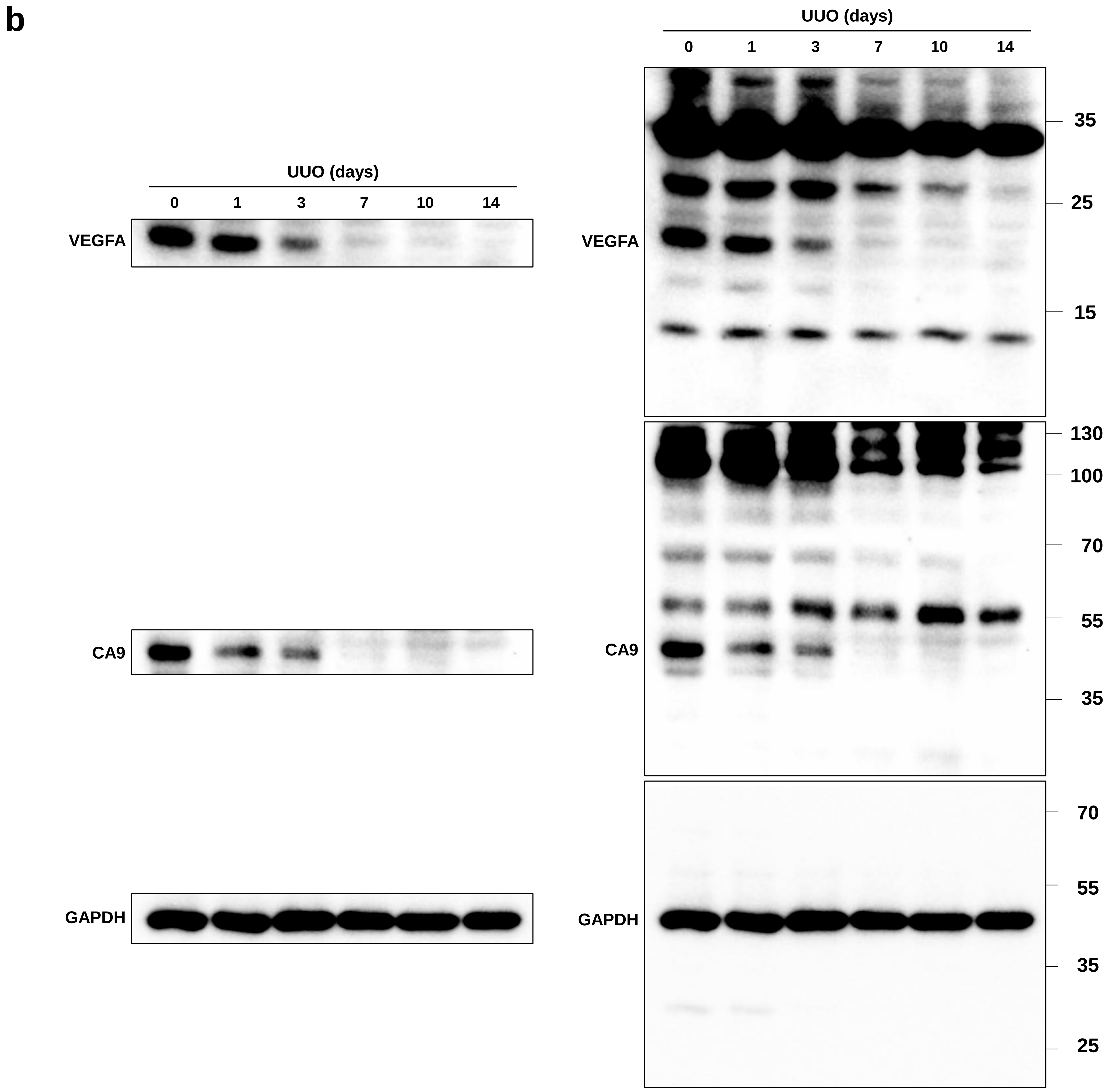
b
UUO (days)
0
1
3
7
10
14
VEGFA
CA9
GAPDH
UUO (days)
0
1
3
7
10
14
VEGFA
35
25
15
CA9
130
100
70
55
35
GAPDH
70
55
35
25

Figure 4

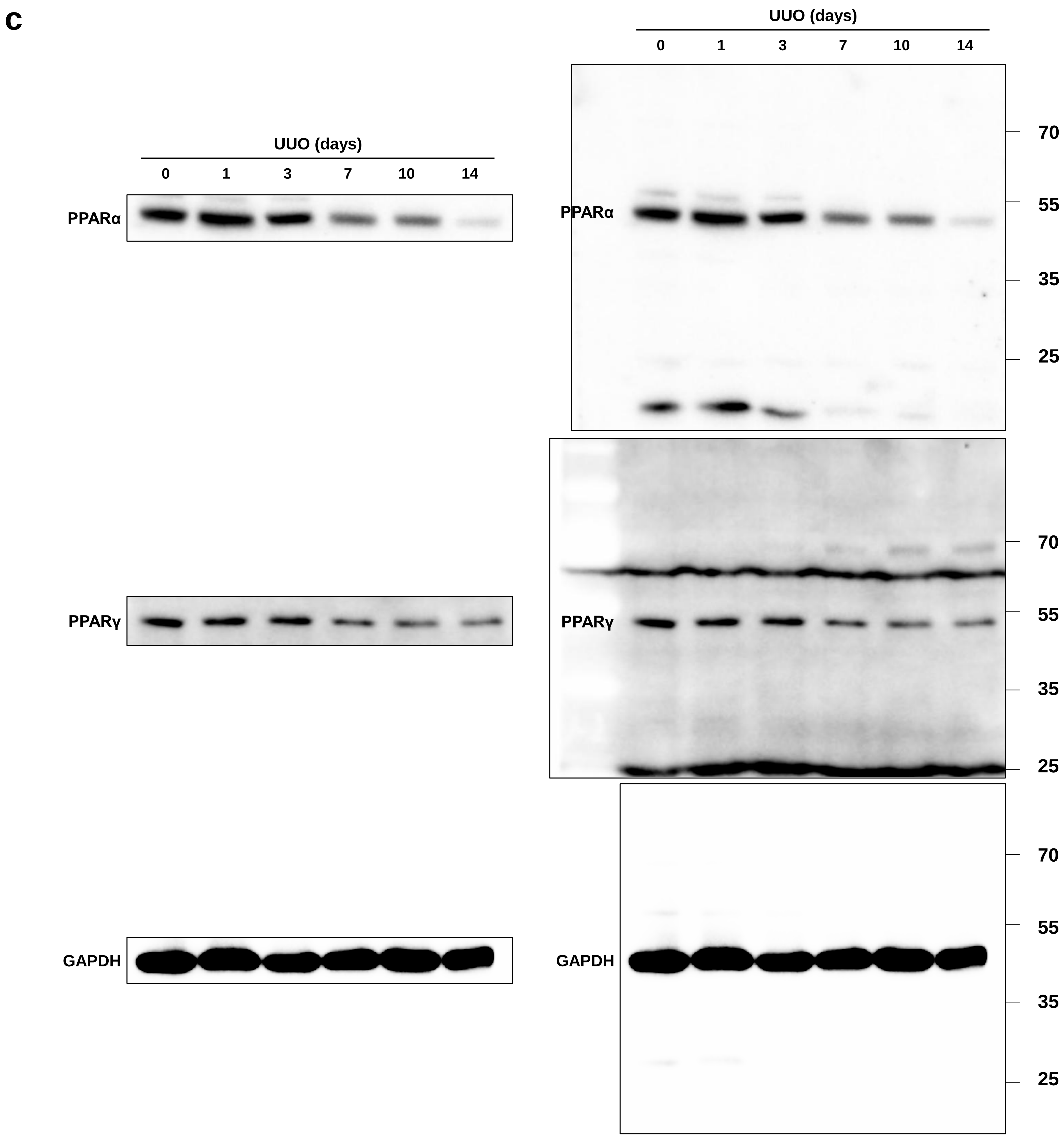
c
UUO (days)
0
1
3
7
10
14
PPARα
PPARγ
GAPDH
UUO (days)
0
1
3
7
10
14
70
55
35
25
PPARα
70
55
35
25
PPARγ
70
55
35
25
GAPDH
