# Supplementary material for: Peroxisomal Degradation Correlates with the Progression of Kidney Injury in a UUO Mouse Model
Source: Biology (Basel). 2026 Jun 25;15(13):996. doi: 10.3390/biology15130996 (PMC13360078; doi:10.3390/biology15130996)
Supplement: Supplementary file 1 [file biology-15-00996-s001.zip › biology-4363317-supplementary.pdf]

Supplementary file

Peroxisomal degradation correlates with the progression of kidney injury in a UUO mouse model

JinHwi Kim[1,2], Hyunsoo Kim[1], Arun Chhetri[1], Laxman Manandhar[1], Gyuho Jang[1], Channy Park[1], and Raekil Park[1*]

[1] Department of biomedical science and engineering, Gwangju Institute of Science and Technology, Gwangju 61005, Korea

nukedata@gist.ac.kr; ruyhyunsookim@gm.gist.ac.kr; chetri5@gm.gist.ac.kr;

laxmanandhar@gm.gist.ac.kr; gyuho9008@gm.gist.ac.kr;

channypark@gist.ac.kr; rkpark@gist.ac.kr

[2] Wonkwang university medical center, Iksan-si, Jeonbuk 54538, Korea; nukedata@gist.ac.kr

* Correspondence: rkpark@gist.ac.kr; Tel.: +82-62-715-5361

Supplementary Table S1. List of antibodies

Antibody	Company	Catalog #	Antibody	Company	Catalog #
anti-4HNE	Abcam	ab46545	anti-GM130	BD bioscience	610822
anti-ACOX1	Proteintech	10957-1-ap	anti-GRP78	Proteintech	14931-1-ap
anti-α-SMA	Abcam	ab124964	anti-HIF1α	R&D system	AF1935
anti-ATG5	Cell Signaling Technology	12994	anti-HIF2α	Novus biologicals	NB100-122
anti-ATG7	Cell Signaling Technology	2631	anti-KIM-1	R&D system	AF1817-SP
anti-Catalase	Abcam	ab16731	anti-LAMP1	Sigma-Aldrich	L1418
anti-collagen I	Southernbiotech	1310-01	anti-LC3B	Sigma-Aldrich	L7543
anti-DBP	Origene	TA308904	anti-Pex14	Santa Cruz	sc-23197
anti-E-cadherin	Proteintech	20874-1-ap	anti-PMP70	Sigma-Aldrich	SAB420018 1
anti-Fibronectin	Abcam	ab2413	anti-VDAC1	Proteintech	55259-1-ap
anti-GAPDH	Cell Signaling Technology	2118	Anti-NBR1	Proteintech	16004-1-AP

Supplementary Table S2. Primer sequences

Primer	Forward	Reverse
36b4	CACTGGTCTAGGACCCGAGAA	GGTGCCTCTGGAGATTTTCG
Gabarap	AAGAGGAGCATCCGTTCGAG	GAGCTTTGGGGGCTTTTTCC
Lc3b	AGATCCCAGTGATTATAGAGCGA	CATGTTCACGTGGTCAGGCA
